# Water table level controls methanogenic and methanotrophic communities and methane emissions in a *Sphagnum*-dominated peatland

**DOI:** 10.1128/spectrum.01992-23

**Published:** 2023-09-25

**Authors:** Wen Tian, Hongmei Wang, Xing Xiang, Prakash C. Loni, Xuan Qiu, Ruicheng Wang, Xianyu Huang, Olli H. Tuovinen

**Affiliations:** 1 State Key Laboratory of Biogeology and Environmental Geology, China University of Geosciences, Wuhan, China; 2 College of Resource and Environment, Anhui Science and Technology of University, Chuzhou, China; 3 Hubei Key Laboratory of Critical Zone Evolution, China University of Geosciences, Wuhan, China; 4 College of Life Science, Shangrao Normal University, Shangrao, China; 5 Department of Microbiology, Ohio State University, Columbus, Ohio, USA; Nanjing Institute of Geography and Limnology Chinese Academy of Sciences, Nanjing, China

**Keywords:** water table level, methanogens, methanotrophs, methane production, methane oxidation, methane fluxes

## Abstract

Peatlands are important sources of the greenhouse gas methane emissions equipoised by methanogens and methanotrophs. However, knowledge about how microbial functional groups associated with methane production and oxidation respond to water table fluctuations has been limited to date. Here, methane-related microbial communities and the potentials of methane production and oxidation were determined along sectioned peat layers in a subalpine peatland across four *Sphagnum*-dominated sites with different water table levels. Methane fluxes were also monitored at these sites. The results showed that *mcrA* gene copies for methanogens were the highest in the 10- to 15-cm peat layer, which was also characterized by the maximum potential methane production (24.53 ± 1.83 nmol/g/h). Copy numbers of the *pmoA* gene for type Ia and Ib methanotrophs were enriched in the 0–5 cm peat layer with the highest potential methane oxidation (43.09 ± 3.44 nmol/g/h). For the type II methanotrophs, the *pmoA* gene copies were higher in the 10- to 15-cm peat layer. Hydrogenotrophic methanogens and type II methanotrophs dominated the methane functional groups. Deterministic process contributed more to methanogenic and methanotrophic community assemblages in comparison with stochastic process. The level of water table significantly shaped methanogenic and methanotrophic community structures and regulated methane fluxes. Compared with vascular plants, *Sphagnum* mosses significantly reduced the methane emissions in peatlands. Collectively, these findings enhance a comprehensive understanding of the effect of the water table level on methane functional groups, with consequential implications for reducing methane emissions within peatland ecosystems.

**IMPORTANCE:**

The water table level is recognized as a critical factor in regulating methane emissions, which are largely dependent on the balance of methanogens and methanotrophs. Previous studies on peat methane emissions have been mostly focused on spatial-temporal variations and the relationship with meteorological conditions. However, the role of the water table level in methane emissions remains unknown. In this work, four representative microhabitats along a water table gradient in a *Sphagnum*-dominated peatland were sampled to gain an insight into methane functional communities and methane emissions as affected by the water table level. The changes in methane-related microbial community structure and assembly were used to characterize the response to the water table level. This study improves the understanding of the changes in methane-related microbial communities and methane emissions with water table levels in peatlands.

## INTRODUCTION

Methane is the second abundant greenhouse gas in the atmosphere. Although the concentration of methane is low, the warming potential of methane is approximately 28 times higher that of carbon dioxide in a 100-year time frame (1). Peatlands store approximately 33% of the terrestrial carbon but with less than 3% land area (2, 3), and thus are considered as enormous carbon sinks. Peatlands are important methane sources due to waterlogging, oxygen deficiencies, and abundant organic matter (4, 5). Peatlands play, therefore, a central role in contributing to global warming and carbon sink-source relationships. Peatland management can be an important means for curtailing methane emissions and mitigating the methane warming effect.

Previous studies have indicated that temperature and vegetation contribute to methane emissions in boreal, tropical, and Qinghai-Tibetan Plateau peatlands (6 – 10). The level of water table has been recently identified as the most important factor in modulating methane emissions (4). The level of water table modifies the redox conditions and oxygen distribution, and alters the composition and succession of vegetation communities. In subalpine peatlands, a long-term change of the water table level can result in successions in vegetation community reassembly (11, 12), thus impacting methane emissions. For example, methane emissions are positively correlated with graminoid plant cover, but the fluxes decrease in sites dominated by shrubs or mosses in peatlands (8, 13). Changes in the water table level may, therefore, indirectly control the methane ﬂuxes by causing changes in other factors, such as vegetation, landscape, and temperature dependence (14, 15).

Net methane emissions in peatlands are determined by the balance between methane production and oxidation associated with methanogens and methanotrophs. Methane formation is strictly anaerobic metabolism by hydrogenotrophic, acetoclastic, methylotrophic, methyl radical, and alkylotrophic methanogens. Hydrogenotrophic and acetoclastic methanogens dominate in paddy fields and freshwater wetlands (16, 17), whereas methylotrophic methanogens are dominant in marine and hypersaline environments (18). The methyl radical process is central in methanogenesis (19). Alkylotrophic methanogens have been detected in sub-surface oil reservoirs (20).

Methanotrophs can be active under both aerobic and anaerobic conditions. Aerobic methanotrophs are phylogenetically clustered into two main clades: type I (*Gammaproteobacteria*, e.g., *Methylococcaceae*), sub-categorized into type Ia and type Ib, and type II (*Alphaproteobacteria*, e,g., *Methylocystaceae*) (21). Type I methanotrophs broadly exist in eutrophic environments, and type II methanotrophs dominate in environments with limited nutrient resources and have high affinity for methane oxidation (22, 23). Anaerobic methanotrophs oxidize methane to carbon dioxide coupled with electron acceptors such as sulfate (24), nitrate (25), nitrite (26), redox metals (27), and humic substances (28).

Methanogens and methanotrophs in peatlands have been studied by molecular techniques, and their distributions and activities are shaped by the water table level (17, 29). In general, methanotrophs are most active in the surface or above the water table due to the oxygen availability (30). Methanogens are relatively active in the transition layer, where the decomposition of organic matter is intense (31, 32). With the drawdown of water table level, the diversity and relative abundance of methanogens decrease, and the methanogenic zone moves deeper into the peat, leading to lesser methane production (32, 33). The diversity of methanotrophs increases, and their relative abundance reaches the maximum at a depth dictated by water table fluctuations (29, 34). The increasing concentration of dissolved organic carbon in rainstorm events or re-wetting conditions, causing increase in the water table level, accelerates the activity of methanogens and methane emissions (35).

Microbial community assembly based on niche and neutral theories is increasingly receiving attention. The niche theory submits that microbial communities are shaped by deterministic processes (36, 37), whereas the neutral theory considers that stochastic processes dominate the microbial community structure (38, 39). Discussions on different opinions and merits on the relative contributions of deterministic and stochastic processes to microbial community assembly are ongoing (40, 41).

The study field, the Dajiuhu peatland, is divided into several patches with water table gradients based on the hydrology and topography. This wetland provides an excellent platform to explore how the water table level affects methane functional groups and methane emissions. The spatial distributions and assemblages of methanogens and methanotrophs were assessed at four *Sphagnum*-dominated sites that had different water table levels. The potentials of methane production and oxidation and methane fluxes were determined at the four sites. This study hypothesized that (i) water table fluctuations alter the structure of methanogenic and methanotrophic communities; (ii) the relative importance of community assembly processes varies with the water table level; and (iii) the level of water table determines methane potential and the spatial variation in methane fluxes.

## MATERIALS AND METHODS

### Site description and sampling

The study area is in the Dajiuhu peatland (31°24′–31°33′N, 109°56′–110°11′E) of Hubei Province, China. A detailed description of the peatland has been published (42). Peat samples were collected in August 2017 from four *Sphagnum*-dominated sites: one each at Erhaoba and Niangniangfen, and two at Yangluchang, each with a different water table level. The water table level was determined with an Odyssey Logger (Dataﬂow Systems Limited, Christchurch) as previously described (43).

At each sampling site, three peat cores were randomly collected using a custom-made sampler (35 cm long and 5 cm wide) and sliced at 5-cm intervals, and then mixed and divided into triplicates. All samples were sealed in sterile tubes and transported in a portable cooler to the geomicrobiology laboratory at China University of Geosciences (Wuhan) within 12 h. Samples from depths 0–5, 10–15, 20–25, and 30–35 cm were analyzed in this study. In total, 48 samples (four depths × four sites × three replicates) were examined. Each sample was divided into two sub-samples: one was stored at 4°C for analysis of physicochemical properties and measurement of methane potential, and the other was kept at −80°C for DNA extraction.

### Potential methane production and oxidation measurements

Measurements of potential methane production and oxidation were carried out as previously described (8). Briefly, ca. 10-g peat samples were added to 100-mL serum bottles. The samples were incubated in the dark at 20°C (the average summer temperature in the study area) for 72 h. Anoxic and oxic conditions were used to determine potential methane production and potential methane oxidation during incubation, respectively.

For the anoxic experiments, the samples in serum bottles were saturated with sterilized peat water to form an anoxic condition and were left without caps in the dark at 20°C. After 24 h, the saturated samples were flushed with N_2_ for 15 min and sealed in a glove box. For the oxic experiments, the samples were left in open serum bottles without caps at 20°C for 24 h. After this acclimation, the serum bottles were sealed with butyl rubber plugs, and 0.5 mL of methane standard (99.999%) was injected using a micro-syringe. Gas samples were collected from the headspace of both anoxic and oxic bottles per 24 h and injected into pre-evacuated vials. Equivalent volumes of N_2_ were injected to the bottles to maintain the gas pressure. Control groups with sterilized peat were included in the anoxic and oxic experiments. The methane concentrations of collected gas samples were analyzed using a model 7890B-5977 gas chromatograph (Agilent, Santa Clara, CA). A known methane standard was also analyzed to check for instrumental error and ensure quality control. The rates of methane production and oxidation were calculated from the linear increases or decreases in the concentration of methane within the bottles over the incubation period after correcting for N_2_ dilution.

### Methane flux measurement

Methane fluxes were measured *in situ* at the four sites using a Los Gatos Research Ultraportable Greenhouse Gas Analyzer (Los Gatos Research, San Jose, CA) and two soil chambers of 30 cm in diameter and 20 cm in height (LICA United Technology Ltd., Beijing) (44). The instrument was calibrated with gas standards before sample measurement. The two chambers were used to monitor methane emissions of (i) bare peat (overlying vegetation was mowed) and (ii) peat covered mainly with *Sphagnum palustre* among the sites, respectively. Methane emissions between *S. palustre* and *Carex* spp. were also analyzed in the first site of Niangniangfen (NNF1). The measurement was one time one site and performed from 11:00 a.m. to 3:00 p.m. with two chambers operated in turn. The data were recorded every 2 min. Methane fluxes were calculated as follows:


$$F=\;\frac{d_{c}}{d_{t}}\;\frac{M}{V_{0}}\;\frac{P}{P_{0}}\;\frac{T_{0}}{T}\;H.$$

where *F* is the methane flux rate; *d*_c_/*d*_t_ is the slope of the linear regression of the methane concentration with time; *M* is the molecular mass of methane; *P* is the air pressure at the sampling site; *T* is the absolute temperature of the sampling time; *V*_0_, *T*_0_, and *P*_0_ are the gas mole volume, absolute temperature, and atmospheric pressure under the standard state condition, respectively; and *H* is the height of the chamber over the water surface or soil surface when there was no water the surface.

### Environmental factors

Peat samples (approximately 10 g) were dried at 50°C for 24 h to measure the water content (WC). The pH, electrical conductivity (EC), total organic carbon (TOC), and total nitrogen (TN) were analyzed as described previously (42). Fresh peat samples (5 g) were sonicated in deionized water (1:4 wt/vol) for 15 min, followed by 20 min of shaking at 200 rpm. The suspension was centrifuged at 5,000 *g* for 5 min and then filtered through a 0.22-µm membrane. The filtrate was analyzed for major anions and cations by a model ICS-1100 ion chromatograph (Thermo Scientific, Waltham, MA) and iCAP-6300 ICP-OES (Thermo Scientific), respectively.

### DNA extraction and *mcrA* and *pmoA* gene amplicon sequencing

DNA was extracted from each sample (0.5-g fresh peat) using a DNeasy PowerSoil Pro Kit (QIAGEN, Düsseldorf, NRW) following the manufacturer’s instructions. DNA quality was determined using a Nanodrop 2000 spectrophotometer (Thermo Scientific). The *mcrA* and *pmoA* genes were amplified using the MLf/MLr (45) and A189f/A650r (46, 47), respectively. Detailed information regarding the PCR amplification of the *mcrA* and *pmoA* genes is available in Table S1.

PCR products were purified with AxyPrep DNA Gel Extraction Kit (Axygen Scientific, Union City, CA) and quantified by FLx800 fluorescence microplate reader (BioTek, Winooski, VT). Sequencing libraries were generated using TruSeq Nano DNA LT Library Prep Kit (Illumina, San Diego, CA) following the manufacturer’s protocols. The acquired libraries were assessed by Agilent 2100 Bioanalyzer and QuantiFluor dsDNA system (Promega, Madison, WI) and then sequenced on an Illumina MiSeq platform with 300-bp paired-end reads (Personalbio, Shanghai).

### Sequence processing

Paired-end raw sequences were performed according to Trimmomatic (48) and assigned to the corresponding samples according to their barcode sequences. Sequences with ambiguous base “N,” quality score <20, and length <150 bp were discarded in a sliding window. High-quality sequences were subsequently merged using FLASH (49) according to the overlap of >10 bp between R1 and R2 reads, and error ratio of the overlap region of <10%. The sequences were checked and corrected by the RDP FrameBot (50), and translated into proteins and mapped to reference protein sequences for further analysis. The high-quality sequences were clustered at 95% cutoﬀ for each operational taxonomic unit (OTU) by UCLUST (51), and singleton OTUs were filtered out. The sequences of mitochondria and chloroplasts were removed, and the chimera sequences were identiﬁed by UCHIME algorithm (52). The reference databases for the *mcrA* and *pmoA* genes were constructed by downloading all *mcrA* and *pmoA* sequences from the Functional Gene Repository (53). The representative sequences for each OTU were annotated referring to the National Center for Biotechnology Information. All samples were then re-sampled to the same level.

### Quantification of *mcrA* and *pmoA* genes

The abundances of *mcrA* and *pmoA* genes in each sample were determined by quantitative PCR (qPCR). The primer pairs mlas/mrcA-rev (54) were used to detect methanogens. To detect type II methanotrophs, the primer set of A189f and A621r (55) was employed. Primer A189f was used with Mb601r and Mc468r to target type Ia and type Ib methanotrophs, respectively (56). Detailed information regarding the qPCR program is available in Table S1.

Standard curves were constructed with 10-fold serially diluted linear pClone007 plasmids (Tsingke Biotechnology, Beijing) containing a targeted fragment from *Escherichia coli* JM109 (Takara Bio, Kusatsu). The ampliﬁcation eﬃciency ranged from 90% to 110%, and the linear ﬁtting showed an *R* ^2^ of >0.99.

### Statistical analysis

The Shannon, Simpson, and Pielou’s evenness indices were calculated, and microbial community dissimilarities were conducted using principal coordinate analysis (PCoA) with Bray-Curtis metric combined permutational multivariate analysis of variance (PERMANOVA). Canonical correspondence analysis (CCA) was used to explore the relationships between environmental variables and microbial communities based on the result of detrended correspondence analysis (57) in the Canoco 5 software. Non-parametric Kruskal-Wallis test was used to compare the differences in physicochemical properties, alpha diversity, taxonomy, and *mcrA* and *pmoA* gene abundances among the samples. Dunnett’s T3 test was used for multiple comparisons between microbial communities collected at diﬀerent sites. Two-way analysis of variance was used to compare methane fluxes among the four sites. Non-parametric Mann-Whitney *U* test was used to discern methane fluxes in two vegetation sites. The regressions between alpha diversity, methane fluxes, and environmental variables were evaluated. Relationships between potential methane production and oxidation, and methanogens and methanotrophs were explored using regression fitting. All statistical analyses were performed using R packages including vegan_2.5–7, ggpubr_0.4.0, and ggplot2_3.4.2 unless stated otherwise.

Networks were constructed for methanogenic and methanotrophic communities based on OTU relative abundances for all samples. The OTUs with relative abundances greater than 0.1% were selected, and a correlation matrix among the OTUs was calculated. Spearman correlation coeﬃcients (*r* >0.7 or *r* <−0.7) with a signiﬁcance of *P* < 0.01 were integrated into the network analysis. Eight topological features of the different networks were estimated by a set of metrics: node number, edge number, average degree, average path length, clustering coefﬁcient, diameter, graph density, and modularity. All analyses were performed with psych_2.1.9 and igraph_1.3.5 packages in R software.

To determine the potential contribution of random processes to community assembly, a neutral community model was constructed. The model evaluates whether the microbial assembly process follows a neutral model (inside model prediction) or a niche-based process (outside model prediction) (38). The parameter *m* describes the migration rate of individual OTU by fitting to the occurrence frequency and relative abundance of OTU across the community in the model. High value of *m* indicates less dispersal limitation in communities and vice versa (36). The parameter *R* ^2^ indicates the degree of fitting in the neutral model. Furthermore, to conﬁrm methanogenic and methanotrophic community assembly processes, the MST was calculated. The MST is an index developed with 50% as the boundary point between more deterministic (<50%) and more stochastic (>50%) assembly (58). Finally, to further explain the pattern of community assembly, the Levin’s niche width was calculated for methane-related microbial communities. These analyses were conducted in R using the Hmisc_4.6–0, NST_3.1.10, and spa_0.2.2 packages.

## RESULTS

### Peat properties

Peat properties in four sites are listed in Table S2. The fluctuation of the water table level (−8.07 to 2.65 cm) affected environmental parameters in peat sediments (Fig. S1). Compared with the NNF1 peat, the the sixth site of Yangluchang (YLC6) peat had higher pH value, WC, TOC, SO_4_^2−^, C:N ratio, and NO_3_^−^ but lower EC, TN, and total Fe. The physicochemical properties of peats also changed with depth (Fig. S1a through i). Together, all peat samples were acidic, with pH ranging from 4.52 to 5.23, and significant differences were observed among the four sites (Fig. S1a, *P* < 0.05). The WC and TOC concentration in all samples were high, and TN concentration was relatively low.

### Methane community diversity and composition

**FIG 1:**
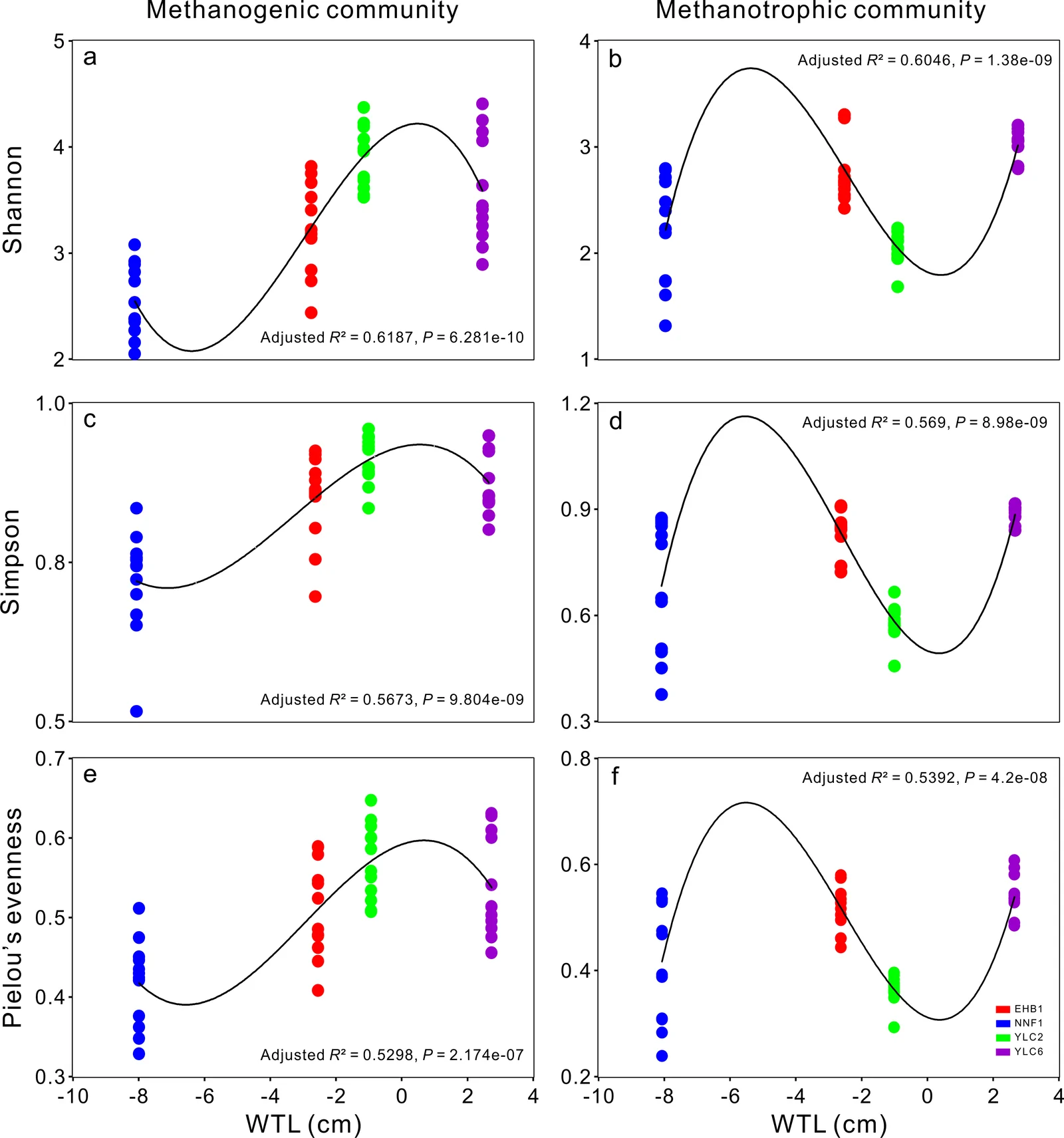
Relationships between the WTL and alpha diversities of methanogens (a, c, and e) and methanotrophs (b, d, and f) from EHB1, NNF1, YLC2, and YLC6 peats. Black solid line indicates fitting curve (*P* < 0.001, *n* = 12). WTL, water table level; EHB1, the first site of Erhaoba; NNF1, the first site of Niangniangfen; YLC2, the second site of Yangluchang; YLC6, the sixth site of Yangluchang.

In 48 peat samples, a total of 3,000,076 and 2,342,335 high-quality *mcrA* and *pmoA* gene sequences were obtained, respectively. The *mcrA* gene sequences were clustered into 3,899 OTUs, and *pmoA* gene sequences were clustered into 1,579 OTUs. The alpha diversity of methane-related microbial communities across different depths in four sites showed significant variations (Fig. S2; Table S3, *P* < 0.05). The alpha diversity indices (Shannon, Simpson, and Pielou’s evenness) of methanogenic and methanotrophic communities showed a significant non-linear correlation with the water table level (Fig. 1, *P* < 0.001), and the two community relationships with the water table level were opposite to each other.

All *mcrA* gene sequences were classiﬁed into 1 phylum, 4 classes, 6 orders, 11 families, and 13 genera (Fig. S3a, c, e). The hydrogenotrophic *Methanomicrobiales* (60.65%) and metabolically diverse *Methanosarcinales* (23.21%) were the dominant orders of methanogenic communities. *Methanoregulaceae* (36.13%), *Methanobacteriaceae* (8.82%), and *Methanocellaceae* (6.77%) belonging to hydrogenotrophic methanogens; *Methanosaetaceae* (13.65%) affiliated with acetoclastic methanogens; and facultative *Methanosarcinaceae* (7.88%) were the dominant families. The hydrogenotrophic *Methanoregula* (29.60%), *Methanobrevibacter* (7.82%), *Methanocella* (6.77%), and *Methanolinea* (2.21%), and facultative *Methanosarcina* (7.88%) were the dominant genera.

All sequences of the *pmoA* gene fell into one phylum, two classes, three orders, four families, and eight genera (Fig. S3b, d, f). The type II methanotroph *Rhizobiales* (74.85%) was the dominant order of methanotrophic communities. *Methylocystaceae* (65.74%) and *Beijerinckiaceae* (9.11%) affiliated with the type II methanotroph were the dominant families. The dominant genera were *Methylosinus* (47.68%), *Methylocystis* (17.05%), and *Methylocapsa* (9.11%) matched into the type II methanotroph. For the type I methanotroph, *Alishewanella* (3.95%) was the dominant genus. Water table fluctuations altered the composition of methanogenic and methanotrophic communities (Tables S4 and S5).

### Functional gene abundances and potential methane production and oxidation

**FIG 2:**
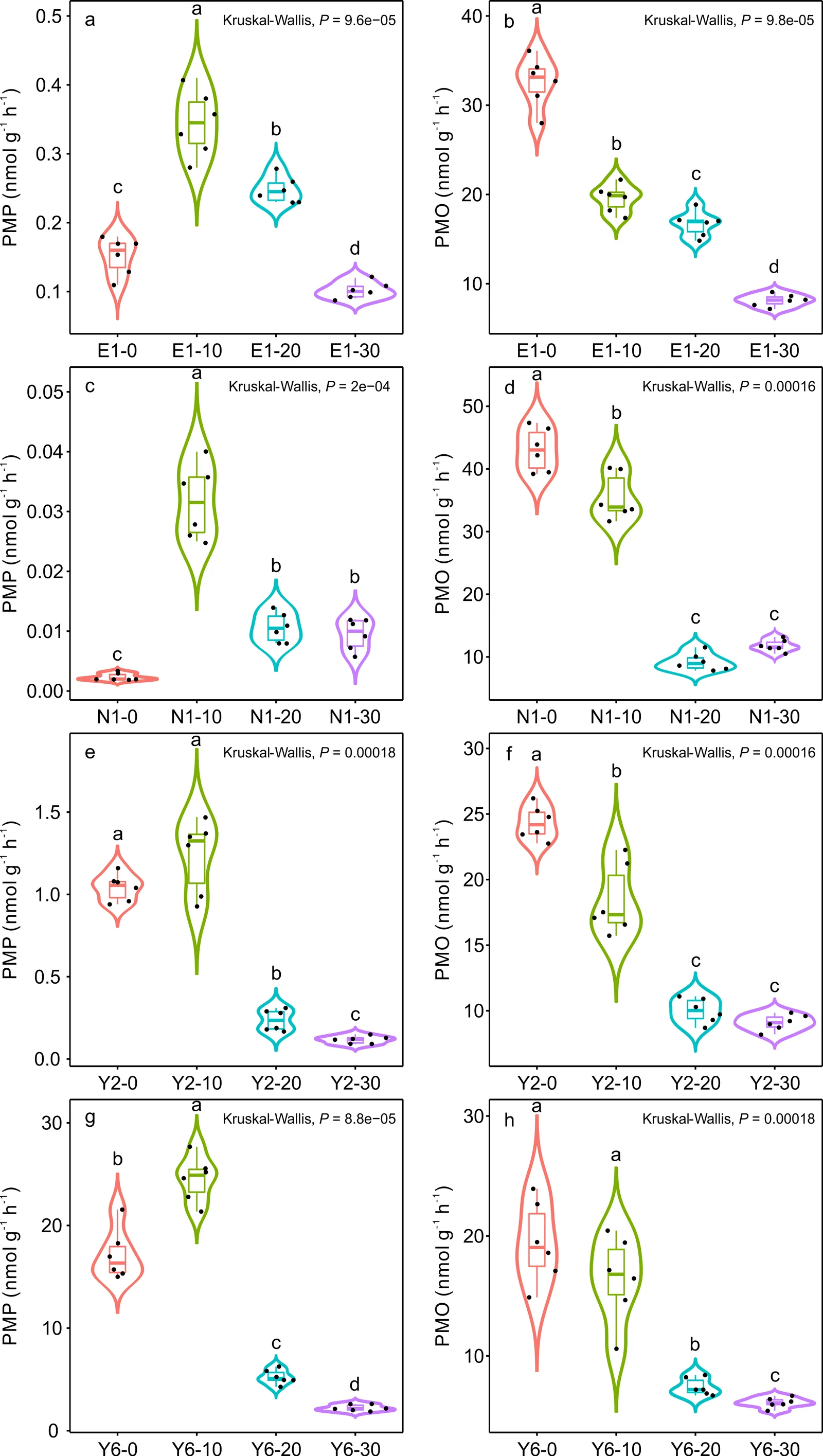
Potential methane production (a, c, e, and g) and oxidation (b, d, f, and h) under anoxic and oxic conditions, respectively, at four depths 0–5, 10–15, 20–25, and 30–35 cm across the four sites. The lowercase letters on each violin plot indicate signiﬁcant difference (*α* = 0.05) based on Dunnett´s T3 test. The same letters indicate no signiﬁcant difference, whereas different letters indicate signiﬁcant difference among depths. E1-0, E1-10, E1-20, and E1-30 represent the depth of 0–5, 10–15, 20–25, and 30–35 cm in EHB1, respectively; N1-0, N1-10, N1-20, and N1-30 represent the depth of 0–5, 10–15, 20–25, and 30–35 cm in NNF1, respectively; Y2-0, Y2-10, Y2-20, and Y2-30 represent the depth of 0–5, 10–15, 20–25, and 30–35 cm in YLC2, respectively; Y6-0, Y6-10, Y6-20, and Y6-30 represent the depth of 0–5, 10–15, 20–25, and 30–35 cm in YLC6, respectively.

**FIG 3:**
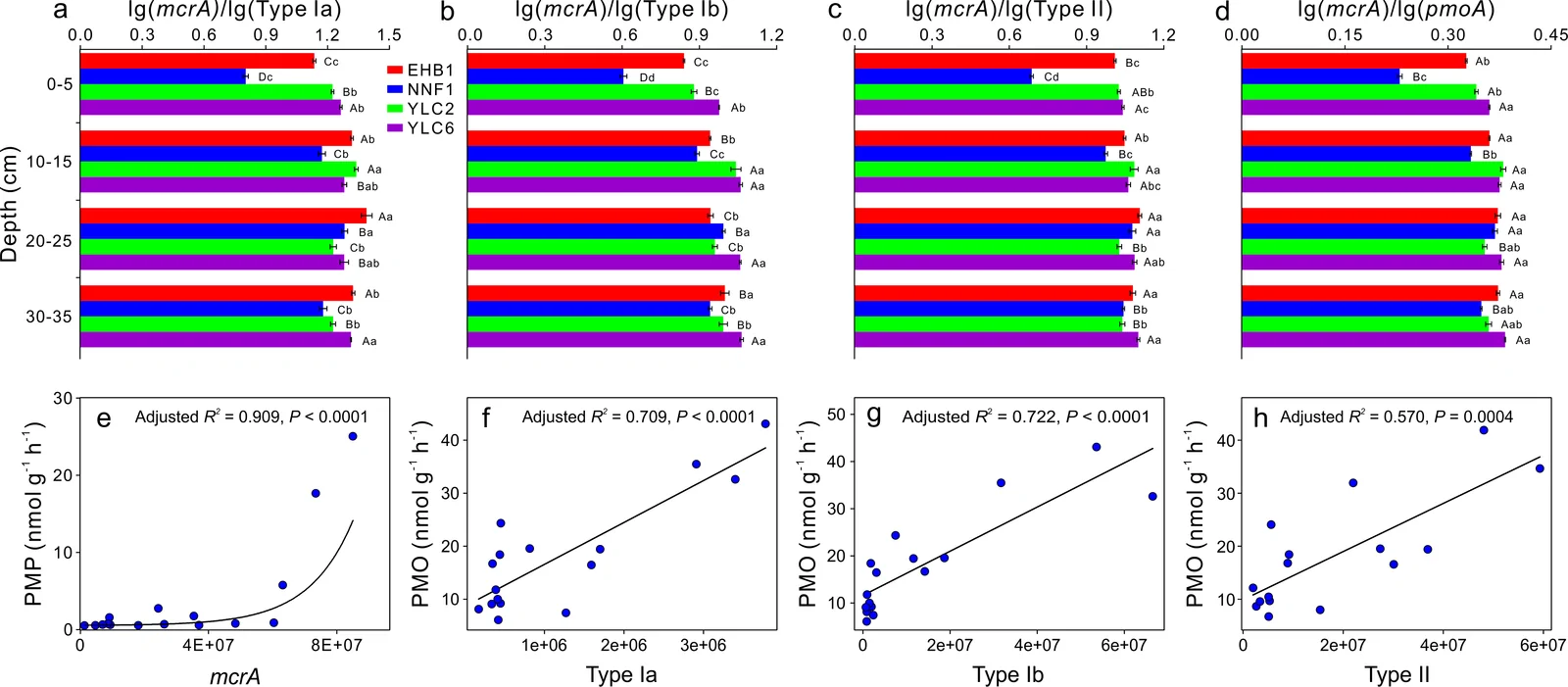
The ratio of *mcrA* to type Ia (a), type Ib (b), type II (c), and *pmoA* (d) among the peats from the four sites. Relationships between the methane potential and the abundance of the *mcrA* (e) and *pmoA* genes including type Ia (f), type Ib (g), and type II (h), respectively. Capital letters mean a statistical significance (*P* < 0.05) among the four sites within the same depth, and small letters mean a statistical significance (*P* < 0.05) among different depths within one site. Black solid line indicates fitting curve (*P* < 0.001, *n* = 16). PMP, potential methane production; PMO, potential methane oxidation.

To examine the abundance of methanogens and methanotrophs in peats, qPCR was used to quantify the *mcrA* and *pmoA* gene copies (Table S6). The *mcrA* gene copy numbers varied from 1.91 × 10^5^ to 8.70 × 10^7^ copies/g fresh peat, and they were the highest in 10- to 15-cm peat layer and then decreased with depth. In line with the *mcrA* gene copies, the highest potential methane production was detected from the 10- to 15-cm peat layer (Fig. 2a, c, e, and g). The potential methane production presented a significant exponential increase with the *mcrA* gene copies (Fig. 3e, *P* < 0.0001).

Of the three methanotrophic types, type II *pmoA* gene copies were generally the highest (2.00 × 10^6^ to 5.93 × 10^7^ copies/g^1^ fresh peat), followed by type Ib *pmoA* (6.81 × 10^5^ to 6.65 × 10^7^ copies/g fresh peat) and type Ia *pmoA* (1.73 × 10^5^ to 3.78 × 10^6^ copies/g fresh peat). For the type II *pmoA*, the highest copy numbers were measured in 10- to 15-cm peat layer except for YLC6 (0–5 cm) and then decreased with depth. Both type Ia and Ib *pmoA* genes had the highest copy numbers in the surface peat (0–5 cm), and they decreased with depth. The ratio of *mcrA* and different *pmoA* types significantly (*P* < 0.05) differed at the four sites (Fig. 3a through d). In line with methanotrophic abundance, the highest potential methane oxidation was measured from the 0- to 5-cm peat layer (Fig. 2b, d, f, and h). The potential methane oxidation correlated positively and significantly with the gene copy numbers of all the three *pmoA* types (Fig. 3f through h, *P* < 0.001).

### Dissimilarity and molecular ecological network of methane communities

**FIG 4:**
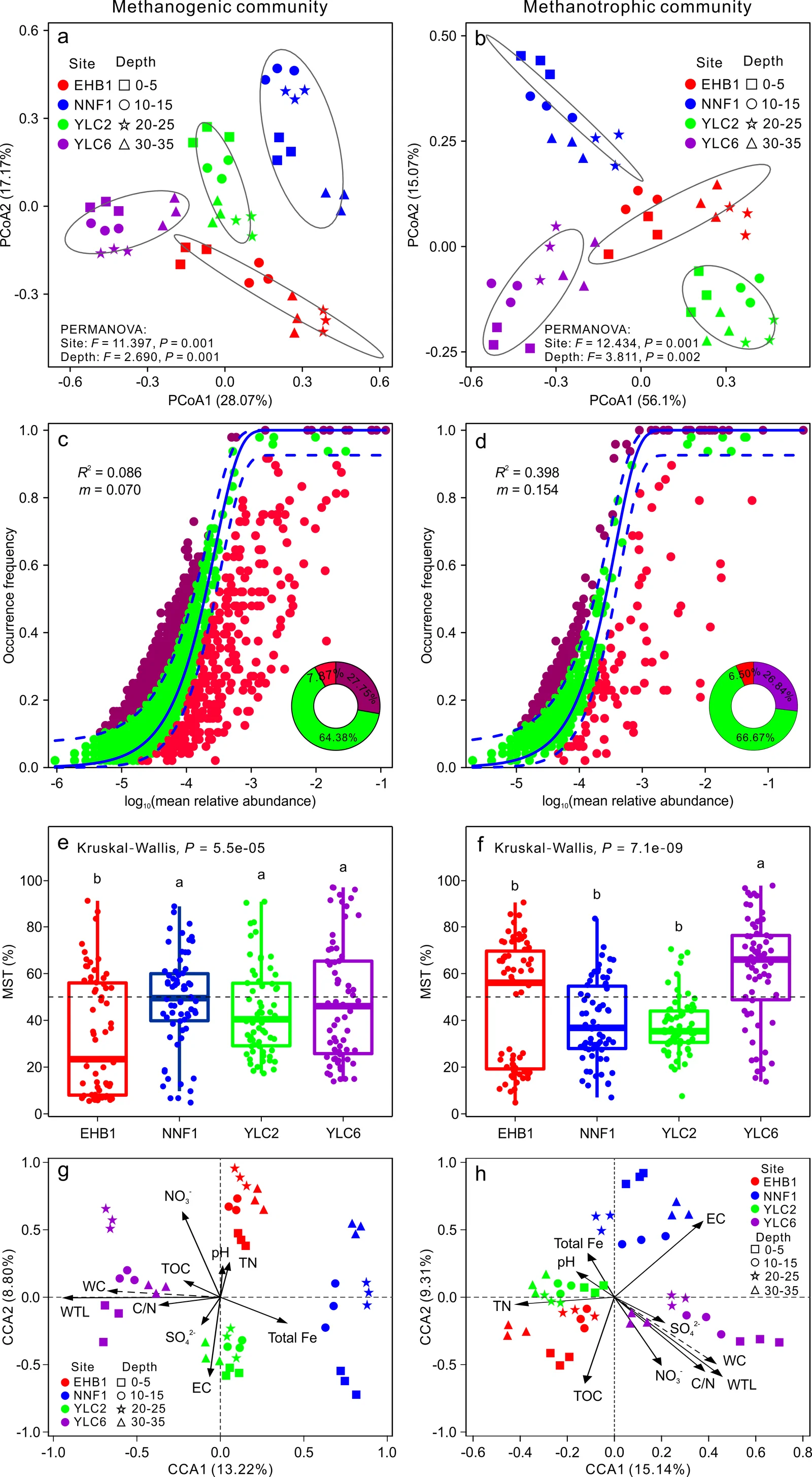
Principal co-ordinate analysis (PCoA) of the methanogenic (a) and methanotrophic (b) community structures. Ellipse shows a 95% conﬁdence interval. The values of PCoA1 and PCoA2 are percentages of explained variations in methanogenic and methanotrophic communities. Colors and shapes represent different sites and depths, respectively. The community dissimilarity values were examined by permutational multivariate analysis of variance (PERMANOVA). Community assembly for the methanogenic (c and e) and methanotrophic (d and f) communities by the neutral community model (NCM) and modified normalized stochasticity ratio (MST). OTUs occurring more frequently than predicted by NCM are shown in purple, while those occurring less frequently than predicted are shown in red. OTUs occurring within prediction are shown in green. The blue solid lines indicate the best fit to the NCM, and the dashed blue lines represent 95% confidence intervals around the model prediction. The MST was developed based on Jaccard distance with 50% as the boundary point between more deterministic (<50%) and more stochastic (>50%) assembly. Nonparametric Kruskal-Wallis test was conducted to test the significance of different sites. Canonical correspondence analysis (CCA) between environmental factors, and methanogens (g) and methanotrophs (h). The values of CCA1 and CCA2 are percentages of explained variations in methanogenic and methanotrophic communities. Colors and shapes represent different sites and depths, respectively. WTL, water table level; EC, electrical conductivity; TN, total nitrogen; TOC, total organic carbon; WC, water content.

The PCoA showed that methanogenic and methanotrophic communities were well segregated across the four sites, indicating that water table fluctuations shifted the structures of peat methanogenic and methanotrophic communities (Fig. 4a and b). Compared with depth, methane-related microbial communities among the sites were significantly much more different as indicated by PERMANOVA.

Co-occurrence networks for methanogenic and methanotrophic communities were constructed to further explore potential microbial interactions. The whole methanotrophic community network appeared to increase its complexity with a shorter average path length and network diameter, higher average degree, clustering coefficient, graph density, and modularity compared with the methanogenic community network (Table 1). Additionally, sub-networks of the four sites in methane-related microbial communities were generated, and a set of topological features was calculated. The node and edge numbers, average degree, clustering coefﬁcient, graph density, and modularity of methanogenic communities were all higher in the the first site of Erhaoba (EHB1), the second site of Yangluchang (YLC2), and YLC6 sub-network than those in the NNF1 sub-network, while the average path length and network diameter were lower. For the methanotrophic communities, the node and edge numbers, average degree, clustering coefﬁcient, and graph density were lower in YLC2 except for the average path length.

**TABLE 1:** Topological features of different co-occurrence networks in peat sediments^*a*^

Category	Node number	Edge number	Average degree	Average path length	Clustering coefficient	Diameter	Graph density	Modularity
Methanogenic community	78	221	5.667	3.666	0.616	9	0.074	0.870
EHB1	68	534	17.706	2.135	0.698	6	0.264	1.803
NNF1	48	168	9.000	2.901	0.666	8	0.191	0.916
YLC2	77	726	20.857	2.226	0.707	6	0.274	5.514
YLC6	76	876	25.053	2.428	0.789	5	0.334	7.823
Methanotrophic community	39	120	6.154	2.259	0.675	6	0.162	1.248
EHB1	49	356	16.531	1.976	0.724	4	0.344	4.374
NNF1	44	273	14.409	2.186	0.718	5	0.335	1.062
YLC2	37	153	10.270	2.339	0.632	6	0.285	4.317
YLC6	41	257	14.537	2.185	0.740	5	0.363	4.502

^*a*^ EHB1, the first site of Erhaoba; NNF1, the first site of Niangniangfen; YLC2, the second site of Yangluchang; YLC6, the sixth site of Yangluchang.

### Community assembly processes and environmental drivers of methane-related microbial communities

The neutral community model (NCM) explained only a small fraction of the relationship between the occurrence frequency of OTUs and their relative abundances for methanogenic (*R* ^2^ = 0.086, *m* = 0.070) and methanotrophic communities (*R* ^2^ = 0.398, *m* = 0.154) (Fig. 4c and d). The *R* ^2^ and *m* values of methanogens were lower as compared to methanotrophs. The proportions of the outlying taxa beyond the blue dashed line were 35.62% and 33.34% for methanogenic and methanotrophic communities, respectively. The dominance test showed that the methane-related microbial community assembly of peats was not well described by neutral-based models. OTUs outside the model prediction accounted for 39.97% and 21.27% of the total sequences in methanogenic and methanotrophic communities, respectively.

The modified normalized stochasticity ratio (MST) based on Jaccard distance showed that the methanogenic (MST = 43.45%) and methanotrophic (MST = 46.40%) communities (Fig. 4e and f; Table S7) in peats were predominately governed by deterministic processes. Compared with the other three sites, the MST of methanogenic communities in EHB1 was significantly lower (Fig. 4e, *P* < 0.001). For methanotrophic communities, stochastic processes played a marginally stronger role in controlling community assembly of YLC6 (Fig. 4f), and determinism dominated in EHB1, NNF1, and YLC2. Pairwise comparisons of MST values for the methanogenic and methanotrophic communities were significantly non-linearly correlated with the water table level (Fig. S4, *P* < 0.001).

CCA was used to understand the relationships between methane-related microbial communities and environmental factors (Fig. 4g and h). CCA explained 53.4% and 50.1% variations of methanogenic and methanotrophic communities, respectively (Table 2). In all the environmental variables, the water table level was the most important factor in driving methane-related community changes. Briefly, the structures of methanogenic and methanotrophic communities in peats were altered from low water table level NNF1 (−8.07 cm) to intermediate water table level EHB1 (−2.63 cm) and YCL2 (−1.01 cm) to high water table level YLC6 (2.65 cm).

**TABLE 2:** Environmental factors correlated to methane microbial communities in peat sediments^*a*^

Factor	Methanogenic community			Methanotrophic community		
	Explained variation (%)	*F*	*P* corrected	Explained variation (%)	*F*	*P* corrected
WTL	12.2	6.4	0.0013**	8.6	4.3	0.0011**
EC	6.9	4.1	0.0013**	5.7	3.4	0.0011**
NO_3_^−^	6.7	3.7	0.0013**	5.0	3.1	0.0011**
Total Fe	5.5	3.5	0.0013**	3.6	2.5	0.0011**
C:N	4.9	3.2	0.0013**	5.5	3.1	0.0011**
pH	4.5	3.1	0.0013**	4.2	2.7	0.0011**
TN	4.2	3.3	0.0013**	4.0	2.7	0.0011**
SO_4_^2−^	4.0	2.9	0.0013**	3.4	2.5	0.0011**
TOC	3.2	2.4	0.0022**	8.4	4.5	0.0011**
WC	1.3	1.0	0.375	1.7	1.3	0.051
Total	53.4	–^*b*^	–	50.1	–	–

^*a*^ WTL, water table level; EC, electrical conductivity; TN, total nitrogen; TOC, total organic carbon; WC, water content. *P* values are adjusted through multiple comparisons using FDR. Bold font represents significant values (α = 0.05); ** stands for significance 0.05 < P** ≤ 0.01.

^*b*^ –, no data.

### Methane fluxes

**FIG 5:**
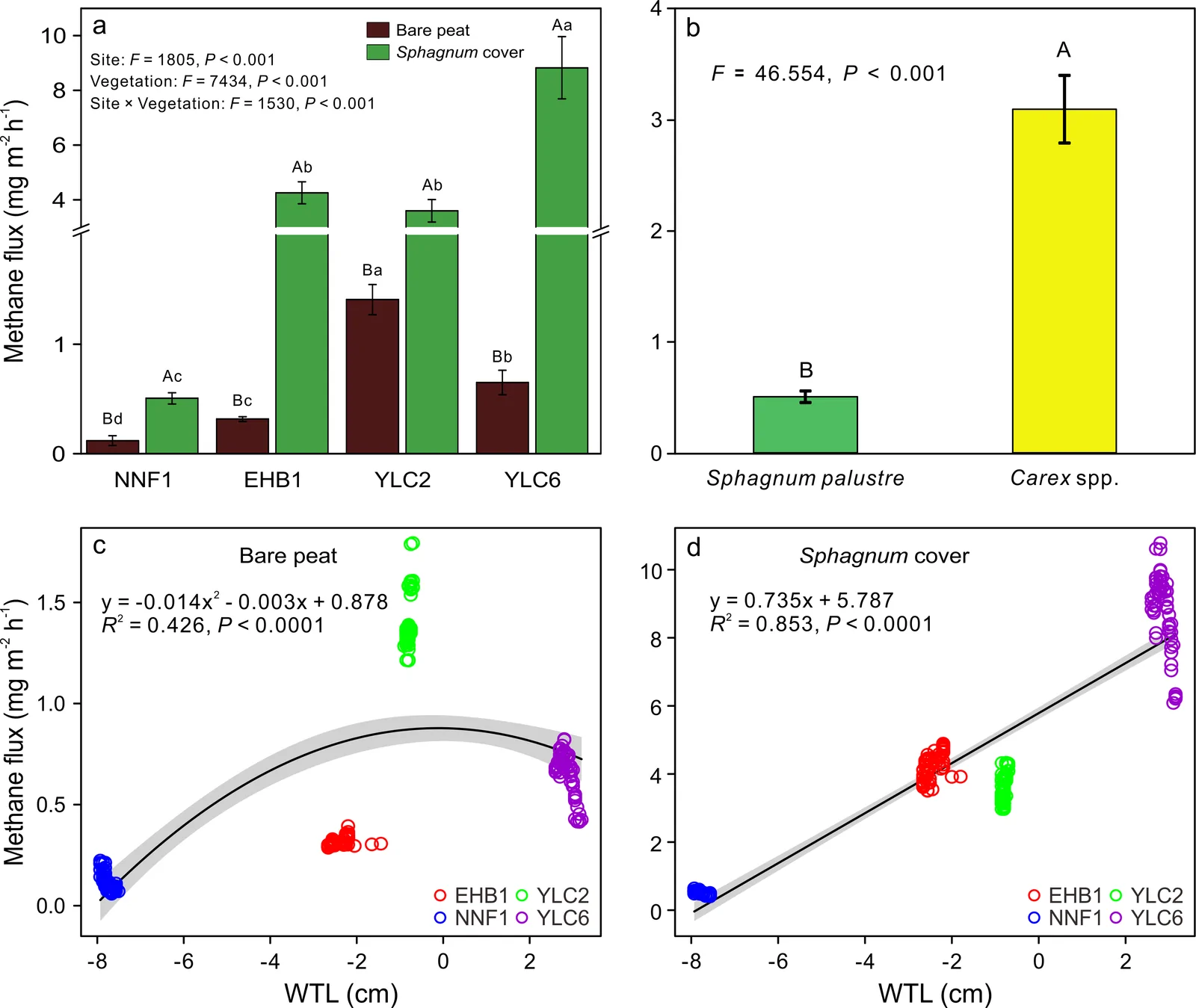
Methane fluxes in bare peat and *Sphagnum* cover across the four sites (a). Different uppercase letters indicate differences between bare peat and *Sphagnum* cover, whereas different lowercase letters indicate differences in bare peat or *Sphagnum* cover among the four sites (Dunnett´s T3 test, *P* < 0.05). Values are means ± SE (*n* = 57). Methane fluxes in the presence of *Sphagnum palustre* and *Carex* spp. (b). Mann-Whitney *U* test was employed to test the significance of difference in methane fluxes. Relationships between water table level and methane fluxes in bare peat (c) and *Sphagnum* cover (d). Black solid line indicates fitting curve (*P* < 0.05, *n* = 57), and gray background represents the 95% conﬁdence level.

Methane fluxes from the four sites with different vegetation types were analyzed (Fig. 5a and b). At each site, methane flux of bare peat was significantly (*P* < 0.05) lower than that of peat covered with *S. palustre*. In bare peat, the YLC2 peat had the highest methane flux (1.41 mg/m^2^/h), followed by the YLC6 (0.65 mg/m^2^/h) and EHB1 (0.32 mg/m^2^/h). The NNF1 peat was the lowest (0.12 mg/m^2^/h). For the *S. palustre* cover peat, the methane flux in YLC6 was significantly (*P* < 0.05) higher than those in the other three sites. Compared to the *Carex* cover peat, methane flux in the *Sphagnum* cover peat significantly (*P* < 0.001) decreased. Furthermore, methane fluxes showed a significant correlation with the water table level in bare and *Sphagnum* cover peats as indicated by a regression model (Fig. 5c and d; *P* < 0.0001).

## DISCUSSION

### The water table level shapes methane communities

Methane-related microbial communities from peat sediments were distinctly diverse among the four sites in the Dajiuhu peatland. Based on the data from the sequencing and quantification of the *mcrA* and *pmoA* genes, the water table level was the strongest factor and significantly shaped the structure of methane-related microbial communities. Methane-related microbial alpha diversity showed a significant non-linear correlation with the increase in the water table level (Fig. 1). The differences in the water table level from the four sites can directly aﬀect methane-related microbial communities by changing physicochemical properties. TOC, C:N ratio, pH, and WC were positively and EC negatively related to the water table level (Fig. S5). Water table fluctuation changes the depth of the oxic-anoxic interface, resulting in shifts in peat decomposability, the bulk density of peat, and electron donors and acceptors (33, 42, 59), consequently affecting the diversity and structures of methanogens and methanotrophs. High water table level facilitates anaerobic metabolism, and low water table level accelerates the aerobic decomposition of organic matter due to the expansion of the oxic zone. Previous studies indicated that long-term peatland drainage increased the thickness and depth of the aerobic layer and promoted the growth of methanotrophs, and the activity of methanogens was inhibited (9, 32, 33, 59). In the present study, the water table level decreased from YLC6 (2.65 cm) to YLC2 (−1.01 cm) to EHB1 (−2.63 cm) to NNF1 (−8.07 cm), suggesting that more oxygen could diffuse into deeper peat layers, thus increasing the thickness of the aerobic layer and changing the structure of methanogenic communities. The structure differentiation was also confirmed in the clustering by PCoA. Type I methanotrophs are mainly distributed in the rhizosphere and topsoil, and type II methanotrophs are dominant in hypoxic regions (60, 61), indicating that the type II methanotrophs are more adaptable to the relatively low oxygen environment compared with the type I methanotrophs. The qPCR results of the *pmoA* gene also showed that the type I methanotrophs were enriched in the surface (0–5 cm), and the type II methanotrophs predominated in the 10- to 15-cm peat layer except for YLC6 (Table S6). The composition of methanotrophic communities was consistent with these variations (Fig. S3). The relative abundance of the type II methanotroph *Rhizobiales* was relatively low in the 0- to 5-cm peat layer, whereas that of the type I methanotroph *Methylococcales* was higher.

### Assembly of methane-related microbial communities

The NCMs, MST index, and niche breadth were used to explain the variations in methane-related microbial communities and associated ecological drivers. The low *R* ^2^ values and <70% species frequency within predicted ranges suggested that the frequency of methanogens and methanotrophs in peats can be less described by the NCMs. Some non-neutral processes should, therefore, be considered. The water table level may be the key factor affecting community assembly, as water bodies potentially help the free movement of microbial communities (36). A low migration rate (*m*) for methanogenic communities and a high *m* for methanotrophic communities in the NCMs implied the signiﬁcant dispersal limitation of methanogens, and high and unhindered dispersal of methanotrophs, respectively. Although some studies suggest that archaea are free to disperse, and thus dispersal limitation is weak (62, 63), other studies submit that archaeal cell sizes are relatively large compared with bacteria, and their dispersal may be limited by environmental sorting (64, 65). In the present study, the migration rate for methanogens was much lower than that for methanotrophs. Peatlands are periodically or permanently flooded environments, and the ﬂow of water would facilitate the movement of methanotrophs. However, the methanogens can be relatively hard to disperse. These results suggested that more methane-related microbial communities are selectively enriched or excluded as a result of water table fluctuation (17, 32, 66).

The MST indices were below 50%, suggesting that the relative importance of deterministic processes was greater than stochastic processes in methane-related microbial communities (Fig. 4e and f; Table S7). The higher environmental heterogeneity in peats expose methanogens and methanotrophs to a greater range of environmental ﬁlters driving the unambiguously deterministic process of environmental selection and causing less community similarity in terms of phylogeny (41, 67). The results implied that the relative effect of stochastic process on structuring methanogenic and methanotrophic communities varied along the water table gradients (Fig. S4), and this was likely related to dispersal limitation proportion. It is possible that dispersal is limited by diminished fluidity in terrestrial ecosystems compared with aquatic ecosystems. Although the high hydrologic connectivity can increase the possibility of the movement of microorganisms, leading to a higher migration rate with the increase of water table level (68), stochastic processes, which are problematic to quantify, may also be altered with water table fluctuations.

The niche-based processes consider that species with wider niche breadth are generalists, which are less inﬂuenced by environmental factors due to the higher environmental tolerances (69). In the present study, the niche breadth of methanotrophs was greater than that of methanogens (Table S8), suggesting that the methanotrophic communities in peats were governed less by environmental ﬁltering. The niche breadth of methane-related microbial communities was positively correlated with stochastic process proportion (Tables S7 and S8). Microbial communities can effectively utilize an array of resources with niche breadth increase (70). Thus, the methanotrophs in peats may be less inﬂuenced by deterministic processes, including environmental ﬁltering, biotic interactions, non-random dispersal, and non-random diversiﬁcation (39) in comparison with methanogens.

### Methane potential and methane fluxes with the water table level

The laboratory incubations showed that potential methane production was positively correlated to the increasing water table level (Fig. S5). Under anaerobic conditions, the type, size, and availability of the substrates were the major factors in methanogenesis. Significantly higher potential methane production was observed in the 10- to 15-cm peat layer (Fig. 2a, c, e, and g) and was likely related to available substrates and anaerobic conditions. Subsequently, potential methane production decreased with the increasing depth of the peat layer as labile substrates were depleted (71). This reduction in potential methane production with depth was reflected in the abundance of *mcrA* gene copies. Potential methane oxidation was negatively related to the increase in the water table level and the ratio of *mcrA*:*pmoA* (Fig. S5). Significantly higher potential methane oxidation was detected in the oxic 0- to 5-cm peat layer (Fig. 2b, d, f, and h), suggesting an increasing effect of the water table level drawdown.

Methane fluxes in bare peat were significantly correlated with the water table level in NNF1 (−8.07 cm), EHB1 (−2.63 cm), YLC2 (−1.01 cm), and YLC6 (2.65 cm), and the lowest emission was recorded in YLC2 (Fig. 5c). Periodic changes of the oxic-anoxic interface with water table fluctuations close to the surface increase availability of electron donors and acceptors, thus facilitating redox reactions (72) in methane cycling. The transport pathway of methane emissions is mainly by ebullition or diffusion in the absence of vegetation (73). Ebullition dominates methane emissions as the water table level above the surface, whereas diffusion is predominant in the upper aerobic layer (74). Ebullition contributes more than diffusion to methane emissions in wetlands, rivers, and lakes (73, 75), consistent with the results of this study.

Vegetation affects methane fluxes in wetlands. Plants provide various substrates including litter, root exudates, and debris, and the quality and quantity of these substrates directly affect microbial metabolism (76, 77). Methane emissions from bare peat and *Sphagnum* covers were divergent across the four sites (Fig. 5a), suggesting that vegetation was the main conduit for methane transport compared to ebullition or molecular diffusion (78). Unlike vascular plants, rootless *Sphagnum* mosses have no ducts and stomata, and grow near the peat surface and usually aggregate into blankets. Methane produced in peats can be oxidized by methanotrophs symbiotic with *Sphagnum* mosses (79 – 81). The symbiosis explains the moderated methane emissions in *Sphagnum*-covered peats as compared to those covered by vascular plants (Fig. 5b), which can directly release 50%–90% methane to the atmosphere via the aerenchyma (74, 82).

Compositionally, methanogens were dominated by the hydrogenotrophs enriched in the 10- to 15-cm peat layer, corresponding to the maximum of methane production. Despite the dominance of the type II methanotrophs in the 10- to 15-cm peat layer, potential methane oxidation rates peaked in the surface (0–5 cm), dominated by the type Ia and Ib methanotrophs. Compared with methanotrophs, community assembly of methanogens was more controlled by the deterministic processes. The relative contribution of different ecological processes may vary with the water table changes. Moreover, the water table level was also the dominant factor impacting the spatial variability in methane fluxes. This peatland study highlights the responses of methanogenic and methanotrophic communities to shifts in the level of water table and lowered methane emissions from *Sphagnum* mosses attributed to methane oxidation by endosymbiotic methanotrophs. These results on methane cycling warrant longer time course and larger scale studies and should be substantiated across different plant types and water table levels for developing peatland management strategies on mitigating methane emissions.

## Data Availability

The raw *pmoA* and *mcrA* gene data are available at the NCBI Sequence Read Archive under BioProject PRJNA884143 and PRJNA884144, respectively.

## SUPPLEMENTAL MATERIAL

Supplemental material spectrum.01992-23-s0001.docx Tables S1 to S8; Fig. S1 to S5.

ASM does not own the copyrights to Supplemental Material that may be linked to, or accessed through, an article. The authors have granted ASM a non-exclusive, world-wide license to publish the Supplemental Material files. Please contact the corresponding author directly for reuse.
