# Supplementary material for: Water table level controls methanogenic and methanotrophic communities and methane emissions in a Sphagnum-dominated peatland
Source: Microbiol Spectr. 2023 Sep 25;11(5):e01992-23. doi: 10.1128/spectrum.01992-23 (PMC10580971; doi:10.1128/spectrum.01992-23)
Supplement: Supplemental material — Tables S1 to S8; Fig. S1 to S5. [file spectrum.01992-23-s0001.docx]

**Table S1** The protocols of PCR reactions in this study

| Target genes | Primer | Sequence 5´-3´ | PCR reaction system | PCR procedure | Reference |
| --- | --- | --- | --- | --- | --- |
| *mcrA*  amplification | MLf | F: GGTGGTGTMGGATTCACACARTAYGCWACAGC | 5 µL 5 × reaction buffer, 5 µL 5 × GC buffer, 2 µL dNTP (2.5 mM), 1 µL each primer (10 mM), 2 µL DNA (20 ng/µL) template, 8.75 µL ddH_2_O and 0.25 µL Q5 DNA Polymerase | An initial denaturation at 98°C for 2 min, 30 cycles of amplification (98°C for 15 s, 55°C for 30 s, 72°C for 30 s) and a final extension at 72°C for 5 min. | 1 |
|  | MLr | R: TTCATTGCRTAGTTWGGRTAGTT |  |  |  |
| *pmoA*  amplification | A189f | F: GGNGACTGGGACTTCTGG |  | 2 min at 98°C for initial denaturation, 30 cycles of 15 s denaturation at 98°C, 20 s annealing at 58°C, and 45 s extension at 72 ℃, followed by 5 min ﬁnal extension at 72 ℃ | 2-3 |
|  | A650r | R: ACGTCCTTACCGAAGGT |  |  |  |
| *mcrA quantification* | mlas | F: GGTGGTGTMGGDTTCACMCARTA | 10 µL of TB Green Premix Ex Taq II (Takara Bio, Japan), 1 µL of each primer (20 µM), 6 µL RNase-free water, 1 µL bovine serum albumin and 1 µL template | Initial denaturation at 95 ℃ for 3 min; 40 cycles denaturation at 95 ℃ for 30 s, annealing at 55 ℃ (methanogens), at 63 ℃ (type II methanotrophs), at 58 ℃(type Ia methanotrophs), at 64 ℃ (type Ib methanotrophs) for 45 s (methanogens), 30 s (type II methanotrophs), 35 s (type Ia and Ib methanotrophs); and extension at 72 ℃ for 30 s (methanogens and type II methanotrophs), 45 s (type Ia and Ib methanotrophs) and ﬁnal extension at 72 ℃ for 10 min | 4 |
|  | mrcA-rev | R: CGTTCATBGCGTAGTTVGGRTAGT |  |  |  |
| type Ia *pmoA quantification* | A189f | F: GGNGACTGGGACTTCTGG |  |  | 2, 5 |
|  | Mb601r | R: ACRTAGTGGTAACCTTGYAA |  |  |  |
| type Ib *pmoA quantification* | A189f | F: GGNGACTGGGACTTCTGG |  |  |  |
|  | A189f | F: GGNGACTGGGACTTCTGG |  |  |  |
|  | Mc468r | R: GCSGTGAACAGGTAGCTGCC |  |  |  |
| type II *pmoA quantification* | A189f | F: GGNGACTGGGACTTCTGG |  |  | 2, 6 |
|  | A621r | R: CGCTCGACCATGCGGAT |  |  |  |

1. Ellis JT, Tramp C, Sims RC, Miller CD. 2012. Characterization of a methanogenic community within an algal fed anaerobic digester. ISRN Microbiol 2012:753892-753903. https://doi.org/10.5402/2012/753892.

2. Holmes AJ, A. Costello A., Lidstrom ME, Murrell JC. 1995. Evidence that particulate methane monooxygenase and ammonia monooxygenase may be evolutionarily related. FEMS Microbiol. Lett. 132:203-208. https://doi.org/10.1111/j.1574-6968.1995.tb07834.x

3. Bourne DG, McDonald IR, Murrell JC. 2001. Comparison of *pmoA* PCR primer sets as tools for investigating methanotroph diversity in three Danish soils. Appl Environ Microbiol 67:3802-3809. https://doi.org/10.1128/AEM.67.9.3802-3809.2001.

4. Steinberg LM, Regan JM. 2008. Phylogenetic comparison of the methanogenic communities from an acidic, oligotrophic fen and an anaerobic digester treating municipal wastewater sludge. Appl Environ Microbiol 74:6663-6671. https://doi.org/10.1128/AEM.00553-08.

5. Kolb S, Knief C, Stubner S, Conrad R. 2003. Quantitative detection of methanotrophs in soil by novel *pmoA*-targeted real-time PCR assays. Appl Environ Microbiol 69:2423-2429. https://doi.org/10.1128/AEM.69.5.2423-2429.2003.

6. Tuomivirta TT, Yrjälä K, Fritze H. 2009. Quantitative PCR of *pmoA* using a novel reverse primer correlates with potential methane oxidation in Finnish fen. Res Microbiol 160:751-756. https://doi.org/10.1016/j.resmic.2009.09.008.

**TABLE S2** Geochemical properties of peat samples from different depths in the four sites at the Dajiuhu Peatland

|  | pH | EC  (μS cm^-1^) | WC  (%) | TOC  (%) | SO_4_^2-^  (mg kg^-1^) | TN  (%) | C/N | Total Fe  (mg kg^-1^) | NO_3_^-^  (mg kg^-1^) | K^+^  (mg kg^-1^) | Na^+^  (mg kg^-1^) | Ca^2+^  (mg kg^-1^) | Mg^2+^  (mg kg^-1^) | Cl^-^  (mg kg^-1^) | WTL  (cm) |
| --- | --- | --- | --- | --- | --- | --- | --- | --- | --- | --- | --- | --- | --- | --- | --- |
| EHB1-0cm-1 | 4.52 | 37.40 | 87.00 | 35.19 | 40.50 | 2.38 | 14.78 | 0.52 | 35.25 | 4.15 | 30.36 | 43.20 | 3.21 | 25.36 | -2.63 |
| EHB1-0cm-2 | 4.53 | 37.30 | 88.60 | 35.18 | 39.75 | 2.42 | 14.51 | 0.43 | 35.47 | 5.32 | 31.23 | 44.00 | 3.01 | 24.67 | -2.63 |
| EHB1-0cm-3 | 4.52 | 37.50 | 87.80 | 34.44 | 38.43 | 2.36 | 14.60 | 0.54 | 34.39 | 4.74 | 34.48 | 39.67 | 2.97 | 27.38 | -2.63 |
| EHB1-10cm-1 | 5.13 | 18.55 | 85.70 | 28.35 | 28.18 | 2.14 | 13.23 | 1.17 | 36.95 | 1.28 | 9.52 | 14.38 | 1.47 | 19.03 | -2.63 |
| EHB1-10cm-2 | 5.03 | 19.39 | 83.70 | 28.19 | 27.01 | 2.11 | 13.35 | 1.23 | 36.44 | 1.54 | 10.03 | 14.93 | 1.44 | 18.93 | -2.63 |
| EHB1-10cm-3 | 5.03 | 19.17 | 84.60 | 28.34 | 27.45 | 2.15 | 13.18 | 1.16 | 35.48 | 1.43 | 11.36 | 15.04 | 1.29 | 18.79 | -2.63 |
| EHB1-20cm-1 | 5.15 | 21.44 | 86.30 | 36.38 | 25.20 | 2.25 | 16.17 | 1.19 | 56.83 | 2.40 | 15.99 | 15.26 | 1.48 | 21.68 | -2.63 |
| EHB1-20cm-2 | 5.07 | 20.93 | 86.60 | 35.70 | 23.47 | 2.27 | 15.76 | 1.25 | 55.94 | 2.53 | 17.06 | 13.65 | 1.34 | 22.46 | -2.63 |
| EHB1-20cm-3 | 5.04 | 20.70 | 85.30 | 36.17 | 24.34 | 2.34 | 15.46 | 1.16 | 56.55 | 2.12 | 14.75 | 15.59 | 1.40 | 21.39 | -2.63 |
| EHB1-30cm-1 | 5.06 | 17.98 | 83.90 | 38.00 | 38.75 | 2.13 | 17.84 | 0.47 | 53.72 | 2.94 | 12.25 | 24.24 | 3.75 | 22.36 | -2.63 |
| EHB1-30cm-2 | 5.08 | 18.07 | 81.70 | 36.43 | 37.55 | 2.17 | 16.78 | 0.54 | 50.29 | 3.03 | 13.59 | 24.06 | 3.49 | 22.00 | -2.63 |
| EHB1-30cm-3 | 5.08 | 17.97 | 81.90 | 35.58 | 38.05 | 2.16 | 16.51 | 0.44 | 51.93 | 2.78 | 12.47 | 23.48 | 3.01 | 21.95 | -2.63 |
| NNF1-0cm-1 | 5.13 | 41.00 | 82.30 | 28.94 | 22.10 | 2.00 | 14.46 | 1.31 | 13.42 | 11.59 | 60.51 | 68.31 | 3.67 | 19.29 | -8.07 |
| NNF1-0cm-2 | 5.13 | 41.40 | 82.10 | 23.28 | 23.55 | 1.91 | 12.17 | 1.43 | 13.02 | 13.24 | 58.39 | 67.01 | 3.75 | 16.73 | -8.07 |
| NNF1-0cm-3 | 5.13 | 41.50 | 81.90 | 22.34 | 22.94 | 1.90 | 11.73 | 1.37 | 12.75 | 10.23 | 63.57 | 66.78 | 3.44 | 18.56 | -8.07 |
| NNF1-10cm-1 | 5.06 | 23.20 | 73.40 | 19.30 | 54.02 | 1.54 | 12.54 | 4.21 | 49.67 | 6.65 | 30.87 | 40.19 | 2.82 | 23.91 | -8.07 |
| NNF1-10cm-2 | 5.09 | 23.30 | 75.60 | 19.14 | 55.74 | 1.58 | 12.13 | 5.85 | 49.03 | 7.34 | 33.67 | 39.74 | 2.67 | 22.54 | -8.07 |
| NNF1-10cm-3 | 5.07 | 23.30 | 73.40 | 18.90 | 53.05 | 1.56 | 12.15 | 4.99 | 47.74 | 6.94 | 29.47 | 40.02 | 2.53 | 22.74 | -8.07 |
| NNF1-20cm-1 | 5.03 | 21.64 | 79.90 | 33.73 | 29.30 | 1.97 | 17.10 | 2.72 | 38.14 | 4.06 | 39.90 | 51.62 | 2.79 | 19.87 | -8.07 |
| NNF1-20cm-2 | 5.00 | 21.60 | 80.40 | 33.36 | 28.02 | 1.91 | 17.43 | 3.57 | 38.72 | 3.99 | 41.93 | 53.05 | 2.54 | 19.53 | -8.07 |
| NNF1-20cm-3 | 4.98 | 21.58 | 78.60 | 33.36 | 28.35 | 1.93 | 17.25 | 3.18 | 40.04 | 4.12 | 38.84 | 50.99 | 2.32 | 20.05 | -8.07 |
| NNF1-30cm-1 | 4.93 | 22.80 | 80.60 | 38.45 | 38.84 | 2.06 | 18.70 | 1.20 | 57.03 | 4.47 | 46.11 | 53.26 | 5.36 | 23.69 | -8.07 |
| NNF1-30cm-2 | 4.96 | 23.20 | 81.40 | 37.58 | 37.54 | 2.05 | 18.32 | 0.88 | 56.14 | 4.27 | 48.76 | 51.24 | 5.04 | 23.14 | -8.07 |
| NNF1-30cm-3 | 4.96 | 23.20 | 81.60 | 35.02 | 36.75 | 1.94 | 18.03 | 1.06 | 55.35 | 4.50 | 44.32 | 54.76 | 4.93 | 22.11 | -8.07 |
| YLC2-0cm-1 | 4.90 | 32.50 | 75.90 | 20.14 | 38.95 | 1.41 | 14.29 | 1.43 | 29.84 | 3.57 | 11.10 | 22.64 | 2.51 | 22.06 | -1.01 |
| YLC2-0cm-2 | 4.89 | 32.00 | 77.34 | 19.96 | 37.54 | 1.39 | 14.39 | 1.38 | 27.55 | 3.47 | 10.02 | 21.03 | 2.34 | 21.02 | -1.01 |
| YLC2-0cm-3 | 4.90 | 31.80 | 78.56 | 20.04 | 38.02 | 1.38 | 14.49 | 1.56 | 29.03 | 3.23 | 12.77 | 23.05 | 2.11 | 22.27 | -1.01 |
| YLC2-10cm-1 | 5.17 | 25.20 | 85.80 | 36.62 | 23.93 | 2.15 | 17.06 | 3.02 | 32.01 | 3.05 | 9.11 | 16.95 | 1.84 | 20.40 | -1.01 |
| YLC2-10cm-2 | 5.15 | 25.20 | 83.80 | 36.75 | 22.12 | 2.19 | 16.76 | 2.74 | 30.06 | 2.85 | 10.01 | 16.05 | 1.64 | 22.12 | -1.01 |
| YLC2-10cm-3 | 5.15 | 25.20 | 84.60 | 36.11 | 23.44 | 2.17 | 16.62 | 2.97 | 31.02 | 3.12 | 9.54 | 17.36 | 1.54 | 21.05 | -1.01 |
| YLC2-20cm-1 | 5.07 | 21.58 | 86.80 | 36.86 | 33.04 | 2.06 | 17.91 | 2.46 | 29.09 | 2.96 | 12.20 | 14.70 | 1.49 | 20.96 | -1.01 |
| YLC2-20cm-2 | 5.08 | 21.69 | 87.50 | 36.25 | 32.97 | 2.10 | 17.23 | 2.09 | 30.00 | 2.88 | 11.34 | 15.04 | 1.12 | 19.05 | -1.01 |
| YLC2-20cm-3 | 5.09 | 21.75 | 86.40 | 34.45 | 31.05 | 2.02 | 17.08 | 1.95 | 28.94 | 3.01 | 10.75 | 14.33 | 1.33 | 19.37 | -1.01 |
| YLC2-30cm-1 | 5.11 | 21.05 | 90.20 | 41.01 | 59.71 | 2.22 | 18.52 | 0.85 | 33.06 | 2.79 | 10.52 | 19.04 | 2.35 | 25.53 | -1.01 |
| YLC2-30cm-2 | 5.10 | 21.09 | 91.35 | 38.19 | 58.84 | 2.27 | 16.82 | 0.59 | 30.53 | 2.56 | 11.55 | 20.38 | 2.13 | 24.06 | -1.01 |
| YLC2-30cm-3 | 5.10 | 21.11 | 89.77 | 41.18 | 60.75 | 2.21 | 18.62 | 0.75 | 31.99 | 2.62 | 10.99 | 18.57 | 2.01 | 26.36 | -1.01 |
| YLC6-0cm-1 | 5.03 | 26.20 | 92.70 | 33.01 | 33.21 | 1.18 | 27.92 | 0.56 | 48.80 | 15.61 | 28.36 | 29.05 | 2.93 | 27.59 | 2.65 |
| YLC6-0cm-2 | 5.08 | 25.10 | 92.30 | 33.37 | 33.76 | 1.25 | 26.74 | 0.54 | 47.06 | 13.47 | 26.73 | 28.89 | 2.57 | 26.53 | 2.65 |
| YLC6-0cm-3 | 5.00 | 25.10 | 90.70 | 38.29 | 44.39 | 1.25 | 30.64 | 0.56 | 46.50 | 14.33 | 26.88 | 30.05 | 2.49 | 27.77 | 2.65 |
| YLC6-10cm-1 | 5.23 | 20.91 | 92.80 | 39.46 | 62.53 | 1.47 | 26.91 | 1.20 | 74.41 | 6.72 | 26.34 | 27.29 | 4.46 | 28.72 | 2.65 |
| YLC6-10cm-2 | 5.20 | 20.65 | 91.40 | 37.34 | 60.53 | 1.49 | 25.07 | 1.38 | 73.66 | 6.54 | 25.77 | 28.73 | 4.31 | 28.05 | 2.65 |
| YLC6-10cm-3 | 5.23 | 20.66 | 90.60 | 38.24 | 63.94 | 1.51 | 25.26 | 1.07 | 71.43 | 7.36 | 25.98 | 26.39 | 4.05 | 29.64 | 2.65 |
| YLC6-20cm-1 | 5.00 | 24.20 | 86.70 | 30.09 | 32.88 | 2.19 | 13.74 | 1.07 | 47.52 | 3.58 | 11.37 | 22.84 | 2.29 | 20.04 | 2.65 |
| YLC6-20cm-2 | 5.04 | 24.00 | 88.30 | 29.10 | 31.12 | 2.20 | 13.20 | 1.16 | 49.54 | 3.78 | 12.74 | 21.88 | 2.12 | 19.99 | 2.65 |
| YLC6-20cm-3 | 5.04 | 24.00 | 86.50 | 30.90 | 33.55 | 2.23 | 13.90 | 0.99 | 48.73 | 3.62 | 10.12 | 20.38 | 1.99 | 21.08 | 2.65 |
| YLC6-30cm-1 | 5.22 | 21.48 | 84.20 | 32.66 | 25.87 | 1.97 | 16.57 | 3.12 | 45.22 | 3.25 | 8.16 | 18.36 | 2.27 | 19.48 | 2.65 |
| YLC6-30cm-2 | 5.17 | 21.57 | 85.20 | 33.08 | 24.05 | 2.01 | 16.43 | 3.40 | 43.58 | 3.42 | 7.47 | 18.00 | 2.04 | 19.00 | 2.65 |
| YLC6-30cm-3 | 5.17 | 21.43 | 82.40 | 34.25 | 26.74 | 2.05 | 16.69 | 3.59 | 44.34 | 3.11 | 8.05 | 17.59 | 1.98 | 20.06 | 2.65 |

EHB1: the first site of Erhaoba; NNF1: the first site of Niangniangfen; YLC2: the second site of Yangluchang; YLC6: the sixth site of Yangluchang; EC: electrical conductivity; WC: water content; TOC: total organic carbon; TN: total nitrogen; C/N: the ratio of total organic carbon to total nitrogen; Total Fe: include Fe^2+^ and Fe^3+^; WTL: water table level. The WTL is expressed with negative number when it is below the peat surface and vice versa.

**TABLE S3** Two-way ANOVA of differences in alpha diversity of methanogenic and methanotrophic communities across the sites and depths

|  | Methanogenic community | | Methanotrophic community | |
| --- | --- | --- | --- | --- |
|  | *F* | *P* | *F* | *P* |
| Site |  |  |  |  |
| Shannon | 103.489 | **0.000** | 94.678 | **0.000** |
| Simpson | 46.012 | **0.000** | 112.925 | **0.000** |
| Pielou’s evenness | 59.682 | **0.000** | 107.926 | **0.000** |
| Depth |  |  |  |  |
| Shannon | 11.590 | **0.000** | 13.189 | **0.000** |
| Simpson | 3.867 | **0.018** | 25.051 | **0.000** |
| Pielou’s evenness | 7.837 | **0.000** | 18.618 | **0.000** |
| Site × Depth |  |  |  |  |
| Shannon | 11.870 | **0.000** | 10.594 | **0.000** |
| Simpson | 5.548 | **0.000** | 13.555 | **0.000** |
| Pielou’s evenness | 11.488 | **0.000** | 17.741 | **0.000** |

Bold font represents significant values (α = 0.05).

**TABLE S4** Kruskal-Wallis test of the relative abundance of methanogenic communities at the order, family and genus levels across the sites and depths

| Taxon | EHB1 | NNF1 | YLC2 | YLC6 |
| --- | --- | --- | --- | --- |
| 0-5 cm | | | | |
| *Methanomicrobiales* | 58.49 ± 1.31ab | 25.97 ± 7.72c | 89.93 ± 9.99a | 30.23 ± 6.96bc |
| *Methanosarcinales* | 28.95 ± 1.43a | 2.45 ± 2.11c | 6.48 ± 1.29b | 24.00 ± 2.55a |
| *Methanobacteriales* | 12.26 ±2.70b | 9.03 ± 7.38bc | 1.40 ± 0.03c | 42.52 ± 7.71a |
| *Methanocellales* | 0.28 ± 0.03c | 62.32 ± 2.22a | 2.14 ± 0.25bc | 2.64 ± 0.65b |
| *Methanomassiliicoccales* | 0.01 ± 0.01a | 0.23 ± 0.20a | 0.06 ± 0.02a | 0.62 ± 0.30a |
| *Methanopyrales* | 0.00 ± 0.00a | 0.00 ± 0.00a | 0.00 ± 0.00a | 0.00 ± 0.00a |
| *Candidatus Methanoperedenaceae* | 0.05 ± 0.00a | 0.00 ± 0.00b | 0.02 ± 0.01ab | 0.00 ± 0.00b |
| *Methanobacteriaceae* | 12.16 ± 2.66b | 9.03 ± 7.38bc | 1.37 ± 2.10c | 38.36 ± 5.73a |
| *Methanocalculaceae* | 0.03 ± 0.01b | 0.00 ± 0.00c | 0.09 ± 0.02a | 0.03 ± 0.01b |
| *Methanocellaceae* | 0.28 ± 0.03c | 62.32 ± 2.22a | 2.14 ± 1.93bc | 2.64 ± 0.65b |
| *Methanocorpusculaceae* | 0.02 ± 0.01a | 0.00 ± 0.00b | 0.03 ± 0.01a | 0.00 ± 0.00b |
| *Methanomassiliicoccaceae* | 0.01 ± 0.01ab | 0.23 ± 0.20a | 0.06 ± 0.08ab | 0.62 ± 0.30a |
| *Methanopyraceae* | 0.00 ± 0.00a | 0.00 ± 0.00a | 0.00 ± 0.00a | 0.00 ± 0.00a |
| *Methanoregulaceae* | 4.52 ± 0.58bc | 11.43 ±5.03b | 33.49 ± 3.87a | 2.96 ± 0.85c |
| *Methanosaetaceae* | 0.71 ± 0.06a | 2.42 ± 2.10ab | 1.57 ± 2.12ab | 0.13 ± 0.01b |
| *Methanosarcinaceae* | 28.19 ± 1.45a | 0.03 ± 0.00b | 4.88 ± 4.17b | 23.86 ± 2.54a |
| *Methanospirillaceae* | 0.00 ± 0.00a | 0.00 ± 0.00a | 0.00 ± 0.00a | 0.00 ± 0.00a |
| *Methanospirillum* | 0.00 ± 0.00a | 0.00 ± 0.00a | 0.00 ± 0.00a | 0.00 ± 0.00a |
| *Methanosphaerula* | 0.00 ± 0.00a | 0.00 ± 0.00a | 0.00 ± 0.00a | 0.00 ± 0.00a |
| *Methanosarcina* | 28.19 ± 1.45a | 0.03 ± 0.00b | 4.88 ± 4.17b | 23.86 ± 2.54a |
| *Methanoregula* | 3.89 ± 0.51c | 10.23 ± 3.96b | 29.11 ± 4.79a | 2.91 ± 0.85c |
| *Methanopyrus* | 0.00 ± 0.00 | 0.00 ± 0.00 | 0.00 ± 0.00 | 0.00 ± 0.00 |
| *Methanomassiliicoccus* | 0.01 ± 0.01b | 0.23 ± 0.20ab | 0.06 ± 0.08b | 0.62 ± 0.30a |
| *Methanolinea* | 0.41 ± 0.11a | 0.65 ± 1.63ab | 2.02 ± 1.48a | 0.03 ± 0.01b |
| *Methanocorpusculum* | 0.02 ± 0.01a | 0.00 ± 0.00b | 0.03 ± 0.01a | 0.00 ± 0.00b |
| *Methanocella* | 0.28 ± 0.03c | 62.32 ± 2.22a | 2.14 ± 1.93bc | 2.64 ± 0.65b |
| *Methanocalculus* | 0.03 ± 0.01b | 0.00 ± 0.00c | 0.09 ± 0.02a | 0.03 ± 0.01b |
| *Methanobrevibacter* | 11.56 ± 2.51b | 6.67 ± 4.13bc | 1.28 ± 1.93c | 35.94 ± 4.85a |
| *Methanobacterium* | 0.60 ± 0.26b | 2.37 ± 3.28ab | 0.10 ± 0.16b | 2.42 ± 0.89a |
| *Candidatus Methanoperedens* | 0.00 ± 0.00a | 0.00 ± 0.00a | 0.00 ± 0.00a | 0.00 ± 0.00a |
| 10-15 cm | | | | |
| *Methanomicrobiales* | 81.84 ± 6.21a | 88.74 ± 1.68a | 96.38 ± 2.82a | 30.61 ± 3.04b |
| *Methanosarcinales* | 16.39 ± 5.81ab | 8.95 ± 1.77b | 2.48 ± 1.75c | 24.90 ± 2.42a |
| *Methanobacteriales* | 0.19 ± 0.23bc | 0.64 ± 0.25b | 0.02 ± 0.01c | 43.33 ± 2.17a |
| *Methanocellales* | 1.51 ± 0.14a | 1.57 ± 0.71a | 1.08 ± 1.05a | 1.11 ± 0.28a |
| *Methanomassiliicoccales* | 0.07 ± 0.07a | 0.09 ± 0.06a | 0.03 ± 0.03a | 0.05 ± 0.03a |
| *Methanopyrales* | 0.00 ± 0.00a | 0.00 ± 0.00a | 0.00 ± 0.00a | 0.00 ± 0.00a |
| *Candidatus Methanoperedenaceae* | 0.06 ± 0.01a | 0.00 ± 0.00c | 0.02 ± 0.01b | 0.00 ± 0.00c |
| *Methanobacteriaceae* | 0.18 ± 0.22bc | 0.57 ± 0.34b | 0.02 ± 0.01c | 42.87 ± 2.32a |
| *Methanocalculaceae* | 0.10 ± 0.06a | 0.13 ± 0.20a | 0.04 ± 0.01a | 0.04 ± 0.01a |
| *Methanocellaceae* | 1.51 ± 0.14a | 1.57 ± 0.71a | 1.08 ± 1.05a | 1.11 ± 0.28a |
| *Methanocorpusculaceae* | 0.01 ± 0.00b | 0.00 ± 0.00c | 0.03 ± 0.00a | 0.00 ± 0.00c |
| *Methanomassiliicoccaceae* | 0.07 ± 0.07a | 0.09 ± 0.06a | 0.03 ± 0.03a | 0.05 ± 0.03a |
| *Methanopyraceae* | 0.00 ± 0.00a | 0.00 ± 0.00a | 0.00 ± 0.00a | 0.00 ± 0.00a |
| *Methanoregulaceae* | 49.91 ± 11.13a | 63.21 ± 2.30a | 55.07 ± 3.98a | 8.86 ± 0.68b |
| *Methanosaetaceae* | 15.85 ± 5.89a | 5.91 ± 0.03b | 1.12 ± 0.31c | 4.08 ± 0.15b |
| *Methanosarcinaceae* | 0.48 ± 0.30c | 3.05 ± 1.75b | 1.34 ± 1.45bc | 20.81 ± 2.56a |
| *Methanospirillaceae* | 0.00 ± 0.00a | 0.00 ± 0.00a | 0.00 ± 0.00a | 0.00 ± 0.00a |
| *Methanospirillum* | 0.00 ± 0.00a | 0.00 ± 0.00a | 0.00 ± 0.00a | 0.00 ± 0.00a |
| *Methanosphaerula* | 0.00 ± 0.00a | 0.00 ± 0.00a | 0.00 ± 0.00a | 0.00 ± 0.00a |
| *Methanosarcina* | 0.48 ± 0.30 | 3.05 ± 1.75 | 1.34 ± 1.45 | 20.81 ± 2.56 |
| *Methanoregula* | 34.49 ± 6.70b | 59.54 ± 2.55a | 49.63 ± 4.09ab | 8.51 ± 0.76c |
| *Methanopyrus* | 0.00 ± 0.00a | 0.00 ± 0.00a | 0.00 ± 0.00a | 0.00 ± 0.00a |
| *Methanomassiliicoccus* | 0.07 ± 0.07a | 0.09 ± 0.06a | 0.03 ± 0.03a | 0.05 ± 0.03a |
| *Methanolinea* | 8.13 ± 12.95ab | 0.28 ± 0.03b | 1.05 ± 0.15a | 0.15 ± 0.11b |
| *Methanocorpusculum* | 0.01 ± 0.01a | 0.00 ± 0.00b | 0.03 ± 0.00a | 0.00 ± 0.00b |
| *Methanocella* | 1.51 ± 0.14 | 1.57 ± 0.71 | 1.08 ± 1.05 | 1.11 ± 0.28 |
| *Methanocalculus* | 1.00 ± 0.06a | 0.13 ± 0.20b | 0.04 ± 0.01b | 0.04 ± 0.01b |
| *Methanobrevibacter* | 0.18 ± 0.21bc | 0.48 ± 0.30b | 0.02 ± 0.01c | 37.23 ± 2.00a |
| *Methanobacterium* | 0.00 ± 0.01c | 0.09 ± 0.07b | 0.00 ± 0.00c | 5.64 ± 0.73a |
| *Candidatus Methanoperedens* | 0.00 ± 0.00a | 0.00 ±0.00a | 0.00 ± 0.00a | 0.00 ± 0.00a |
| 20-25 cm | | | | |
| *Methanomicrobiales* | 38.93 ± 6.67b | 93.51 ± 4.85a | 84.18 ± 3.77a | 39.63 ± 10.69b |
| *Methanosarcinales* | 54.30 ± 5.69a | 4.24 ± 3.28c | 12.04 ± 2.53c | 31.62 ± 4.30b |
| *Methanobacteriales* | 0.04 ± 0.01b | 0.08 ± 0.05b | 0.09 ± 0.04b | 27.79 ± 8.65a |
| *Methanocellales* | 6.73 ± 1.11a | 2.16 ± 1.64bc | 3.58 ± 1.28bc | 0.89 ± 0.28c |
| *Methanomassiliicoccales* | 0.01 ± 0.00b | 0.00 ± 0.00b | 0.04 ± 0.06ab | 0.07 ± 0.02a |
| *Methanopyrales* | 0.00 ± 0.00b | 0.00 ± 0.00b | 0.06 ± 0.05a | 0.00 ± 0.00b |
| *Candidatus Methanoperedenaceae* | 0.04 ± 0.01a | 0.04 ± 0.07a | 0.22 ± 0.12a | 0.00 ± 0.00b |
| *Methanobacteriaceae* | 0.04 ± 0.01b | 0.08 ± 0.05b | 0.09 ± 0.03b | 27.75 ± 8.63a |
| *Methanocalculaceae* | 0.00 ± 0.00a | 0.01 ± 0.03a | 0.01 ± 0.01a | 0.00 ± 0.00a |
| *Methanocellaceae* | 6.73 ± 1.11a | 2.16 ± 1.64ab | 3.58 ± 1.28ab | 0.89 ± 0.28b |
| *Methanocorpusculaceae* | 0.12 ± 0.03a | 0.00 ± 0.00b | 0.27 ± 0.12a | 0.00 ± 0.00b |
| *Methanomassiliicoccaceae* | 0.01 ± 0.00a | 0.00 ± 0.00b | 0.04 ± 0.06ab | 0.07 ± 0.02a |
| *Methanopyraceae* | 0.00 ± 0.00b | 0.00 ± 0.00b | 0.06 ± 0.05a | 0.00 ± 0.00b |
| *Methanoregulaceae* | 31.28 ± 7.28b | 83.55 ± 11.29a | 61.80 ± 2.59a | 26.99 ± 8.24b |
| *Methanosaetaceae* | 54.06 ± 5.70a | 2.74 ± 1.91c | 10.28 ± 2.27b | 6.15 ± 2.74bc |
| *Methanosarcinaceae* | 0.20 ± 0.01c | 1.46 ± 1.33bc | 1.54 ± 0.40b | 25.47 ± 6.23a |
| *Methanospirillaceae* | 0.00 ± 0.00b | 0.00 ± 0.00b | 0.50 ± 0.35a | 0.00 ± 0.00b |
| *Methanospirillum* | 0.00 ± 0.00b | 0.00 ± 0.00b | 0.50 ± 0.35a | 0.00 ± 0.00b |
| *Methanosphaerula* | 0.00 ± 0.00b | 0.00 ± 0.00b | 0.03 ± 0.01a | 0.00 ± 0.00b |
| *Methanosarcina* | 0.20 ± 0.01c | 1.46 ± 1.33bc | 1.54 ± 0.40b | 25.47 ± 6.23a |
| *Methanoregula* | 23.29 ± 5.95b | 73.10 ± 14.99a | 38.04 ± 1.33b | 25.58 ± 7.89b |
| *Methanopyrus* | 0.00 ± 0.00b | 0.00 ± 0.00b | 0.06 ± 0.05a | 0.00 ± 0.00b |
| *Methanomassiliicoccus* | 0.01 ± 0.00b | 0.00 ± 0.00b | 0.04 ± 0.06ab | 0.07 ± 0.02a |
| *Methanolinea* | 2.10 ± 0.89a | 6.10 ± 5.21a | 2.45 ± 0.67a | 0.15 ± 0.03b |
| *Methanocorpusculum* | 0.12 ± 0.03a | 0.00 ± 0.00b | 0.27 ± 0.12a | 0.00 ± 0.00b |
| *Methanocella* | 6.73 ± 1.11a | 2.16 ± 1.64ab | 3.58 ± 1.28ab | 0.89 ± 0.28b |
| *Methanocalculus* | 0.00 ± 0.00a | 0.01 ± 0.03a | 0.01 ± 0.01a | 0.00 ± 0.00a |
| *Methanobrevibacter* | 0.04 ± 0.01b | 0.07 ± 0.04b | 0.08 ± 0.03b | 25.03 ± 8.11a |
| *Methanobacterium* | 0.00 ± 0.00b | 0.01 ± 0.01b | 0.00 ± 0.00b | 2.72 ± 0.53a |
| *Candidatus Methanoperedens* | 0.00 ± 0.00b | 0.00 ± 0.00b | 0.09 ± 0.02a | 0.00 ± 0.00b |
| 30-35 cm | | | | |
| *Methanomicrobiales* | 42.43 ± 14.94ab | 37.00 ± 2.39b | 69.04 ± 6.62a | 63.46 ± 4.55a |
| *Methanosarcinales* | 50.95 ± 11.99a | 55.92 ± 2.34a | 18.90 ± 0.58c | 28.77 ± 0.99b |
| *Methanobacteriales* | 0.11 ± 0.06b | 0.04 ± 0.01c | 1.74 ± 1.53ab | 6.71 ± 5.13a |
| *Methanocellales* | 6.44 ± 2.98a | 7.03 ± 0.40a | 8.21 ± 3.64a | 0.58 ± 0.34b |
| *Methanomassiliicoccales* | 0.06 ± 0.02b | 0.00 ± 0.00c | 1.91 ± 1.05a | 0.48 ± 0.40a |
| *Methanopyrales* | 0.00 ± 0.00b | 0.00 ± 0.0b | 0.20 ± 0.18a | 0.00 ± 0.00b |
| *Candidatus Methanoperedenaceae* | 17.48 ± 2.58a | 0.04 ± 0.02c | 8.89 ± 2.69b | 0.00 ± 0.01c |
| *Methanobacteriaceae* | 0.11 ± 0.06b | 0.04 ± 0.01b | 1.72 ± 1.52ab | 6.66 ± 5.10a |
| *Methanocalculaceae* | 0.00 ± 0.00b | 0.00 ± 0.00b | 0.02 ± 0.01a | 0.00 ± 0.00b |
| *Methanocellaceae* | 6.44 ± 2.98a | 7.03 ± 0.40a | 8.21 ± 3.64a | 0.58 ± 0.34b |
| *Methanocorpusculaceae* | 0.05 ± 0.02b | 0.02 ± 0.03b | 1.54 ± 1.07a | 0.00 ± 0.00c |
| *Methanomassiliicoccaceae* | 0.06 ± 0.02b | 0.00 ± 0.00c | 1.91 ± 1.05a | 0.48 ± 0.40a |
| *Methanopyraceae* | 0.00 ± 0.00b | 0.00 ± 0.00b | 0.20 ± 0.18a | 0.00 ± 0.00b |
| *Methanoregulaceae* | 30.21 ± 10.60ab | 31.50 ± 1.99b | 34.49 ± 2.88ab | 48.86 ± 6.32a |
| *Methanosaetaceae* | 32.46 ± 10.91a | 55.03 ± 2.66a | 6.49 ± 1.01b | 19.36 ± 4.58b |
| *Methanosarcinaceae* | 1.01 ± 0.26b | 0.85 ± 0.41b | 3.52 ± 2.27ab | 9.40 ± 4.04a |
| *Methanospirillaceae* | 0.00 ± 0.00b | 0.00 ± 0.00b | 0.81 ± 0.57a | 0.00 ± 0.00b |
| *Methanospirillum* | 0.00 ± 0.00b | 0.00 ± 0.00b | 0.81 ± 0.57a | 0.00 ± 0.00b |
| *Methanosphaerula* | 0.00 ± 0.00b | 0.00 ± 0.00b | 0.06 ± 0.05a | 0.00 ± 0.00b |
| *Methanosarcina* | 1.01 ± 0.26 | 0.85 ± 0.41 | 3.52 ± 2.27 | 9.40 ± 4.04 |
| *Methanoregula* | 22.29 ± 8.61b | 28.04 ± 0.00b | 23.16 ± 4.56b | 41.80 ± 6.48a |
| *Methanopyrus* | 0.00 ± 0.00b | 0.00 ± 0.00b | 0.20 ± 0.18a | 0.00 ± 0.00b |
| *Methanomassiliicoccus* | 0.06 ± 0.02b | 0.00 ± 0.00c | 1.91 ± 1.05a | 0.48 ± 0.40ab |
| *Methanolinea* | 2.32 ± 0.31b | 1.98 ± 0.97b | 6.45 ± 1.32a | 1.17 ± 0.42b |
| *Methanocorpusculum* | 0.05 ± 0.02b | 0.02 ± 0.02b | 1.54 ± 1.07a | 0.00 ± 0.00c |
| *Methanocella* | 6.44 ± 2.98a | 7.03 ± 0.40a | 8.21 ± 3.64a | 0.58 ± 0.34b |
| *Methanocalculus* | 0.00 ± 0.00b | 0.00 ± 0.00b | 0.02 ± 0.01a | 0.00 ± 0.00b |
| *Methanobrevibacter* | 0.11 ± 0.06b | 0.04 ± 0.01b | 1.55 ± 1.37a | 4.83 ± 3.87a |
| *Methanobacterium* | 0.00 ± 0.00b | 0.01 ± 0.01b | 0.17 ± 0.16a | 1.84 ± 1.27a |
| *Candidatus Methanoperedens* | 0.07 ± 0.01a | 0.00 ± 0.00b | 1.02 ± 0.28a | 0.00 ± 0.00b |
| Taxon | 0-5 cm | 10-15 cm | 20-25 cm | 30-35 cm |
| EHB1 |  |  |  |  |
| *Methanomicrobiales* | 58.49 ± 1.31b | 81.84 ± 6.21a | 38.93 ± 6.67c | 42.43 ± 14.94bc |
| *Methanosarcinales* | 28.95 ± 1.43b | 16.39 ± 5.81c | 54.30 ± 5.69a | 50.95 ± 11.99a |
| *Methanobacteriales* | 12.26 ±2.70a | 0.19 ± 0.23b | 0.04 ± 0.01b | 0.11 ± 0.06b |
| *Methanocellales* | 0.28 ± 0.03c | 1.51 ± 0.14b | 6.73 ± 1.11a | 6.44 ± 2.98a |
| *Methanomassiliicoccales* | 0.01 ± 0.01a | 0.07 ± 0.07a | 0.01 ± 0.00a | 0.06 ± 0.02a |
| *Methanopyrales* | 0.00 ± 0.00a | 0.00 ± 0.00a | 0.00 ± 0.00a | 0.00 ± 0.00a |
| *Candidatus Methanoperedenaceae* | 0.05 ± 0.00b | 0.06 ± 0.01b | 0.04 ± 0.01b | 17.48 ± 2.58a |
| *Methanobacteriaceae* | 12.16 ± 2.66a | 0.18 ± 0.22b | 0.04 ± 0.01b | 0.11 ± 0.06b |
| *Methanocalculaceae* | 0.03 ± 0.01a | 0.10 ± 0.06a | 0.00 ± 0.00b | 0.00 ± 0.00b |
| *Methanocellaceae* | 0.28 ± 0.03c | 1.51 ± 0.14b | 6.73 ± 1.11a | 6.44 ± 2.98a |
| *Methanocorpusculaceae* | 0.02 ± 0.01b | 0.01 ± 0.00b | 0.12 ± 0.03a | 0.05 ± 0.02ab |
| *Methanomassiliicoccaceae* | 0.01 ± 0.01a | 0.07 ± 0.07a | 0.01 ± 0.00a | 0.06 ± 0.02a |
| *Methanopyraceae* | 0.00 ± 0.00a | 0.00 ± 0.00a | 0.00 ± 0.00a | 0.00 ± 0.00a |
| *Methanoregulaceae* | 4.52 ± 0.58b | 49.91 ± 11.13a | 31.28 ± 7.28a | 30.21 ± 10.60a |
| *Methanosaetaceae* | 0.71 ± 0.06c | 15.85 ± 5.89b | 54.06 ± 5.70a | 32.46 ± 10.91b |
| *Methanosarcinaceae* | 28.19 ± 1.45a | 0.48 ± 0.30b | 0.20 ± 0.01b | 1.01 ± 0.26b |
| *Methanospirillaceae* | 0.00 ± 0.00a | 0.00 ± 0.00a | 0.00 ± 0.00a | 0.00 ± 0.00a |
| *Methanospirillum* | 0.00 ± 0.00a | 0.00 ± 0.00a | 0.00 ± 0.00a | 0.00 ± 0.00a |
| *Methanosphaerula* | 0.00 ± 0.00a | 0.00 ± 0.00a | 0.00 ± 0.00a | 0.00 ± 0.00a |
| *Methanosarcina* | 28.19 ± 1.45a | 0.48 ± 0.30b | 0.20 ± 0.01b | 1.01 ± 0.26b |
| *Methanoregula* | 3.89 ± 0.51b | 34.49 ± 6.70a | 23.29 ± 5.95a | 22.29 ± 8.61a |
| *Methanopyrus* | 0.00 ± 0.00a | 0.00 ± 0.00a | 0.00 ± 0.00a | 0.00 ± 0.00a |
| *Methanomassiliicoccus* | 0.01 ± 0.01a | 0.07 ± 0.07a | 0.01 ± 0.00a | 0.06 ± 0.02a |
| *Methanolinea* | 0.41 ± 0.11b | 8.13 ± 12.95ab | 2.10 ± 0.89a | 2.32 ± 0.31a |
| *Methanocorpusculum* | 0.02 ± 0.01b | 0.01 ± 0.01b | 0.12 ± 0.03a | 0.05 ± 0.02ab |
| *Methanocella* | 0.28 ± 0.03c | 1.51 ± 0.14b | 6.73 ± 1.11a | 6.44 ± 2.98a |
| *Methanocalculus* | 0.03 ± 0.01 | 1.00 ± 0.06 | 0.00 ± 0.00 | 0.00 ± 0.00 |
| *Methanobrevibacter* | 11.56 ± 2.51a | 0.18 ± 0.21b | 0.04 ± 0.01b | 0.11 ± 0.06b |
| *Methanobacterium* | 0.60 ± 0.26a | 0.00 ± 0.01b | 0.00 ± 0.00b | 0.00 ± 0.00b |
| *Candidatus Methanoperedens* | 0.00 ± 0.00b | 0.00 ± 0.00b | 0.00 ± 0.00b | 0.07 ± 0.01a |
| NNF1 |  |  |  |  |
| *Methanomicrobiales* | 25.97 ± 7.72b | 88.74 ± 1.68a | 93.51 ± 4.85a | 37.00 ± 2.39b |
| *Methanosarcinales* | 2.45 ± 2.11b | 8.95 ± 1.77b | 4.24 ± 3.28b | 55.92 ± 2.34a |
| *Methanobacteriales* | 9.03 ± 7.38a | 0.64 ± 0.25a | 0.08 ± 0.05b | 0.04 ± 0.01b |
| *Methanocellales* | 62.32 ± 2.22a | 1.57 ± 0.71c | 2.16 ± 1.64c | 7.03 ± 0.40b |
| *Methanomassiliicoccales* | 0.23 ± 0.20a | 0.09 ± 0.06a | 0.00 ± 0.00b | 0.00 ± 0.00b |
| *Methanopyrales* | 0.00 ± 0.00a | 0.00 ± 0.00a | 0.00 ± 0.00a | 0.00 ± 0.00a |
| *Candidatus Methanoperedenaceae* | 0.00 ± 0.00a | 0.00 ± 0.00a | 0.04 ± 0.07a | 0.04 ± 0.02a |
| *Methanobacteriaceae* | 9.03 ± 7.38a | 0.57 ± 0.34a | 0.08 ± 0.05b | 0.04 ± 0.01b |
| *Methanocalculaceae* | 0.00 ± 0.00 | 0.13 ± 0.20a | 0.01 ± 0.03a | 0.00 ± 0.00a |
| *Methanocellaceae* | 62.32 ± 2.22a | 1.57 ± 0.71c | 2.16 ± 1.64c | 7.03 ± 0.40b |
| *Methanocorpusculaceae* | 0.00 ± 0.00a | 0.00 ± 0.00a | 0.00 ± 0.00a | 0.02 ± 0.03a |
| *Methanomassiliicoccaceae* | 0.23 ± 0.20a | 0.09 ± 0.06a | 0.00 ± 0.00b | 0.00 ± 0.00b |
| *Methanopyraceae* | 0.00 ± 0.00a | 0.00 ± 0.00a | 0.00 ± 0.00a | 0.00 ± 0.00a |
| *Methanoregulaceae* | 11.43 ±5.03c | 63.21 ± 2.30a | 83.55 ± 11.29a | 31.50 ± 1.99b |
| *Methanosaetaceae* | 2.42 ± 2.10c | 5.91 ± 0.03b | 2.74 ± 1.91c | 55.03 ± 2.66a |
| *Methanosarcinaceae* | 0.03 ± 0.00b | 3.05 ± 1.75a | 1.46 ± 1.33ab | 0.85 ± 0.41b |
| *Methanospirillaceae* | 0.00 ± 0.00a | 0.00 ± 0.00a | 0.00 ± 0.00a | 0.00 ± 0.00a |
| *Methanospirillum* | 0.00 ± 0.00a | 0.00 ± 0.00a | 0.00 ± 0.00a | 0.00 ± 0.00a |
| *Methanosphaerula* | 0.00 ± 0.00a | 0.00 ± 0.00a | 0.00 ± 0.00a | 0.00 ± 0.00a |
| *Methanosarcina* | 0.03 ± 0.00b | 3.05 ± 1.75a | 1.46 ± 1.33a | 0.85 ± 0.41a |
| *Methanoregula* | 10.23 ± 3.96c | 59.54 ± 2.55a | 73.10 ± 14.99a | 28.04 ± 0.00b |
| *Methanopyrus* | 0.00 ± 0.00a | 0.00 ± 0.00a | 0.00 ± 0.00a | 0.00 ± 0.00a |
| *Methanomassiliicoccus* | 0.23 ± 0.20a | 0.09 ± 0.06a | 0.00 ± 0.00b | 0.00 ± 0.00b |
| *Methanolinea* | 0.65 ± 1.63a | 0.28 ± 0.03a | 6.10 ± 5.21a | 1.98 ± 0.97a |
| *Methanocorpusculum* | 0.00 ± 0.00a | 0.00 ± 0.00a | 0.00 ± 0.00a | 0.02 ± 0.02a |
| *Methanocella* | 62.32 ± 2.22a | 1.57 ± 0.71c | 2.16 ± 1.64c | 7.03 ± 0.40b |
| *Methanocalculus* | 0.00 ± 0.00a | 0.13 ± 0.20a | 0.01 ± 0.03a | 0.00 ± 0.00a |
| *Methanobrevibacter* | 6.67 ± 4.13a | 0.48 ± 0.30b | 0.07 ± 0.04b | 0.04 ± 0.01b |
| *Methanobacterium* | 2.37 ± 3.28a | 0.09 ± 0.07a | 0.01 ± 0.01a | 0.01 ± 0.01a |
| *Candidatus Methanoperedens* | 0.00 ± 0.00a | 0.00 ±0.00a | 0.00 ± 0.00a | 0.00 ± 0.00a |
| YLC2 |  |  |  |  |
| *Methanomicrobiales* | 89.93 ± 9.99ab | 96.38 ± 2.82a | 84.18 ± 3.77ab | 69.04 ± 6.62b |
| *Methanosarcinales* | 6.48 ± 1.29b | 2.48 ± 1.75c | 12.04 ± 2.53ab | 18.90 ± 0.58a |
| *Methanobacteriales* | 1.40 ± 0.03a | 0.02 ± 0.01b | 0.09 ± 0.04b | 1.74 ± 1.53ab |
| *Methanocellales* | 2.14 ± 0.25b | 1.08 ± 1.05b | 3.58 ± 1.28ab | 8.21 ± 3.64a |
| *Methanomassiliicoccales* | 0.06 ± 0.02b | 0.03 ± 0.03b | 0.04 ± 0.06b | 1.91 ± 1.05a |
| *Methanopyrales* | 0.00 ± 0.00b | 0.00 ± 0.00b | 0.06 ± 0.05a | 0.20 ± 0.18a |
| *Candidatus Methanoperedenaceae* | 0.02 ± 0.01c | 0.02 ± 0.01c | 0.22 ± 0.12b | 8.89 ± 2.69a |
| *Methanobacteriaceae* | 1.37 ± 2.10ab | 0.02 ± 0.01b | 0.09 ± 0.03ab | 1.72 ± 1.52a |
| *Methanocalculaceae* | 0.09 ± 0.02a | 0.04 ± 0.01ab | 0.01 ± 0.01b | 0.02 ± 0.01b |
| *Methanocellaceae* | 2.14 ± 1.93a | 1.08 ± 1.05a | 3.58 ± 1.28a | 8.21 ± 3.64a |
| *Methanocorpusculaceae* | 0.03 ± 0.01b | 0.03 ± 0.00b | 0.27 ± 0.12a | 1.54 ± 1.07a |
| *Methanomassiliicoccaceae* | 0.06 ± 0.08b | 0.03 ± 0.03b | 0.04 ± 0.06b | 1.91 ± 1.05a |
| *Methanopyraceae* | 0.00 ± 0.00b | 0.00 ± 0.00b | 0.06 ± 0.05a | 0.20 ± 0.18a |
| *Methanoregulaceae* | 33.49 ± 3.87b | 55.07 ± 3.98a | 61.80 ± 2.59a | 34.49 ± 2.88b |
| *Methanosaetaceae* | 1.57 ± 2.12b | 1.12 ± 0.31c | 10.28 ± 2.27a | 6.49 ± 1.01ab |
| *Methanosarcinaceae* | 4.88 ± 4.17a | 1.34 ± 1.45a | 1.54 ± 0.40a | 3.52 ± 2.27a |
| *Methanospirillaceae* | 0.00 ± 0.00b | 0.00 ± 0.00b | 0.50 ± 0.35a | 0.81 ± 0.57a |
| *Methanospirillum* | 0.00 ± 0.00b | 0.00 ± 0.00b | 0.50 ± 0.35a | 0.81 ± 0.57a |
| *Methanosphaerula* | 0.00 ± 0.00b | 0.00 ± 0.00b | 0.03 ± 0.01a | 0.06 ± 0.05a |
| *Methanosarcina* | 4.88 ± 4.17a | 1.34 ± 1.45a | 1.54 ± 0.40a | 3.52 ± 2.27a |
| *Methanoregula* | 29.11 ± 4.79b | 49.63 ± 4.09a | 38.04 ± 1.33ab | 23.16 ± 4.56c |
| *Methanopyrus* | 0.00 ± 0.00b | 0.00 ± 0.00b | 0.06 ± 0.05a | 0.20 ± 0.18a |
| *Methanomassiliicoccus* | 0.06 ± 0.08b | 0.03 ± 0.03b | 0.04 ± 0.06b | 1.91 ± 1.05a |
| *Methanolinea* | 2.02 ± 1.48b | 1.05 ± 0.15b | 2.45 ± 0.67b | 6.45 ± 1.32a |
| *Methanocorpusculum* | 0.03 ± 0.01b | 0.03 ± 0.00b | 0.27 ± 0.12a | 1.54 ± 1.07a |
| *Methanocella* | 2.14 ± 1.93ab | 1.08 ± 1.05b | 3.58 ± 1.28ab | 8.21 ± 3.64a |
| *Methanocalculus* | 0.09 ± 0.02a | 0.04 ± 0.01ab | 0.01 ± 0.01b | 0.02 ± 0.01b |
| *Methanobrevibacter* | 1.28 ± 1.93ab | 0.02 ± 0.01b | 0.08 ± 0.03ab | 1.55 ± 1.37a |
| *Methanobacterium* | 0.10 ± 0.16ab | 0.00 ± 0.00b | 0.00 ± 0.00b | 0.17 ± 0.16a |
| *Candidatus Methanoperedens* | 0.00 ± 0.00c | 0.00 ± 0.00c | 0.09 ± 0.02b | 1.02 ± 0.28a |
| YLC6 |  |  |  |  |
| *Methanomicrobiales* | 30.23 ± 6.96b | 30.61 ± 3.04b | 39.63 ± 10.69b | 63.46 ± 4.55a |
| *Methanosarcinales* | 24.00 ± 2.55a | 24.90 ± 2.42a | 31.62 ± 4.30a | 28.77 ± 0.99a |
| *Methanobacteriales* | 42.52 ± 7.71a | 43.33 ± 2.17a | 27.79 ± 8.65a | 6.71 ± 5.13b |
| *Methanocellales* | 2.64 ± 0.65a | 1.11 ± 0.28b | 0.89 ± 0.28b | 0.58 ± 0.34b |
| *Methanomassiliicoccales* | 0.62 ± 0.30a | 0.05 ± 0.03b | 0.07 ± 0.02b | 0.48 ± 0.40ab |
| *Methanopyrales* | 0.00 ± 0.00a | 0.00 ± 0.00a | 0.00 ± 0.00a | 0.00 ± 0.00a |
| *Candidatus Methanoperedenaceae* | 0.00 ± 0.00a | 0.00 ± 0.00a | 0.00 ± 0.00a | 0.00 ± 0.01a |
| *Methanobacteriaceae* | 38.36 ± 5.73a | 42.87 ± 2.32a | 27.75 ± 8.63a | 6.66 ± 5.10b |
| *Methanocalculaceae* | 0.03 ± 0.01a | 0.04 ± 0.01a | 0.00 ± 0.00b | 0.00 ± 0.00b |
| *Methanocellaceae* | 2.64 ± 0.65a | 1.11 ± 0.28b | 0.89 ± 0.28b | 0.58 ± 0.34b |
| *Methanocorpusculaceae* | 0.00 ± 0.00a | 0.00 ± 0.00a | 0.00 ± 0.00a | 0.00 ± 0.00a |
| *Methanomassiliicoccaceae* | 0.62 ± 0.30a | 0.05 ± 0.03b | 0.07 ± 0.02b | 0.48 ± 0.40ab |
| *Methanopyraceae* | 0.00 ± 0.00a | 0.00 ± 0.00a | 0.00 ± 0.00a | 0.00 ± 0.00a |
| *Methanoregulaceae* | 2.96 ± 0.85d | 8.86 ± 0.68c | 26.99 ± 8.24b | 48.86 ± 6.32a |
| *Methanosaetaceae* | 0.13 ± 0.01c | 4.08 ± 0.15b | 6.15 ± 2.74b | 19.36 ± 4.58a |
| *Methanosarcinaceae* | 23.86 ± 2.54a | 20.81 ± 2.56a | 25.47 ± 6.23a | 9.40 ± 4.04b |
| *Methanospirillaceae* | 0.00 ± 0.00a | 0.00 ± 0.00a | 0.00 ± 0.00a | 0.00 ± 0.00a |
| *Methanospirillum* | 0.00 ± 0.00a | 0.00 ± 0.00a | 0.00 ± 0.00a | 0.00 ± 0.00a |
| *Methanosphaerula* | 0.00 ± 0.00a | 0.00 ± 0.00a | 0.00 ± 0.00a | 0.00 ± 0.00a |
| *Methanosarcina* | 23.86 ± 2.54a | 20.81 ± 2.56a | 25.47 ± 6.23a | 9.40 ± 4.04b |
| *Methanoregula* | 2.91 ± 0.85c | 8.51 ± 0.76b | 25.58 ± 7.89a | 41.80 ± 6.48a |
| *Methanopyrus* | 0.00 ± 0.00a | 0.00 ± 0.00a | 0.00 ± 0.00a | 0.00 ± 0.00a |
| *Methanomassiliicoccus* | 0.62 ± 0.30a | 0.05 ± 0.03b | 0.07 ± 0.02b | 0.48 ± 0.40ab |
| *Methanolinea* | 0.03 ± 0.01c | 0.15 ± 0.11bc | 0.15 ± 0.03b | 1.17 ± 0.42a |
| *Methanocorpusculum* | 0.00 ± 0.00a | 0.00 ± 0.00a | 0.00 ± 0.00a | 0.00 ± 0.00a |
| *Methanocella* | 2.64 ± 0.65a | 1.11 ± 0.28b | 0.89 ± 0.28b | 0.58 ± 0.34b |
| *Methanocalculus* | 0.03 ± 0.01a | 0.04 ± 0.01a | 0.00 ± 0.00b | 0.00 ± 0.00b |
| *Methanobrevibacter* | 35.94 ± 4.85a | 37.23 ± 2.00a | 25.03 ± 8.11a | 4.83 ± 3.87b |
| *Methanobacterium* | 2.42 ± 0.89b | 5.64 ± 0.73a | 2.72 ± 0.53b | 1.84 ± 1.27b |
| *Candidatus Methanoperedens* | 0.00 ± 0.00a | 0.00 ± 0.00a | 0.00 ± 0.00a | 0.00 ± 0.00a |

Values are means ± standard error (n = 3). Lowercase letters in the same row indicate significant difference (α = 0.05) based on Dunn’s multiple comparison tests.

**TABLE S5** Kruskal-Wallis test of the relative abundance of methanotrophic communities at the order, family and genus levels across the sites and depths

| Taxon | EHB1 | NNF1 | YLC2 | YLC6 |
| --- | --- | --- | --- | --- |
| 0-5 cm | | | | |
| *Rhizobiales* | 50.64 ± 0.84b | 86.92 ± 2.82a | 87.18 ± 2.97a | 30.74 ± 2.07c |
| *Alteromonadales* | 5.51 ± 0.28a | 3.39 ± 0.17 | 2.59 ± 0.16 | 4.40 ± 0.14a |
| *Methylococcales* | 29.18 ± 1.33b | 5.89 ± 2.04c | 0.95 ± 0.38d | 35.90 ± 0.87a |
| *Methylocystaceae* | 37.35 ± 1.76b | 43.03 ± 6.17b | 81.63 ± 2.83a | 21.45 ± 2.61c |
| *Methylococcaceae* | 0.00 ± 0.00b | 0.09 ± 0.16a | 0.59 ± 0.21a | 0.00 ± 0.00b |
| *Beijerinckiaceae* | 13.29 ± 1.22b | 43.90 ± 3.39a | 5.55 ± 5.64b | 9.29 ± 1.41b |
| *Alteromonadaceae* | 5.51 ± 0.28a | 3.39 ± 0.17c | 2.59 ± 0.16d | 4.40 ± 0.14b |
| *Methylosarcina* | 0.00 ± 0.00a | 0.00 ± 0.00a | 0.00 ± 0.00a | 0.00 ± 0.00a |
| *Methyloparacoccus* | 0.00 ± 0.00b | 0.09 ± 0.16ab | 0.43 ± 0.38a | 0.00 ± 0.00b |
| *Candidatus Methylospira* | 0.00 ± 0.00a | 0.00 ± 0.00a | 0.16 ± 0.17a | 0.00 ± 0.00a |
| *Alishewanella* | 5.51 ± 0.28a | 3.39 ± 0.17c | 2.59 ± 0.16d | 4.40 ± 0.14b |
| *Methylosinus* | 10.71 ± 1.08b | 16.72 ± 6.52b | 72.75 ± 2.80a | 3.84 ± 1.00c |
| *Methylocystis* | 25.49 ± 1.72a | 25.92 ± 1.99a | 7.83 ± 0.13c | 17.23 ± 1.70b |
| *Methylocapsa* | 13.29 ± 1.22b | 43.90 ± 3.39a | 5.55 ± 5.64b | 9.29 ± 1.41b |
| 10-15 cm | | | | |
| *Rhizobiales* | 77.07 ± 2.29b | 70.80 ± 13.62ab | 92.89 ± 0.69a | 45.99 ± 1.76c |
| *Alteromonadales* | 6.40 ± 0.58a | 5.41 ± 0.68a | 3.13 ± 0.14b | 5.85 ± 0.53a |
| *Methylococcales* | 0.47 ± 0.13b | 14.94 ± 17.19ab | 1.15 ± 0.62b | 22.32 ± 2.56a |
| *Methylocystaceae* | 74.02 ± 3.79b | 61.06 ± 11.90b | 87.88 ± 1.63a | 37.04 ± 3.05c |
| *Methylococcaceae* | 0.00 ± 0.01a | 0.03 ± 0.05a | 0.00 ± 0.00a | 0.01 ± 0.01a |
| *Beijerinckiaceae* | 3.05 ± 1.57a | 9.74 ± 5.20a | 5.01 ± 1.99a | 8.95 ± 1.76a |
| *Alteromonadaceae* | 6.40 ± 0.58a | 5.41 ± 0.68a | 3.13 ± 0.14b | 5.85 ± 0.53a |
| *Methylosarcina* | 0.00 ± 0.01a | 0.00 ± 0.00a | 0.00 ± 0.00a | 0.00 ± 0.00a |
| *Methyloparacoccus* | 0.00 ± 0.00a | 0.00 ± 0.00a | 0.00 ± 0.00a | 0.00 ± 0.00a |
| *Candidatus Methylospira* | 0.00 ± 0.00a | 0.03 ± 0.05a | 0.00 ± 0.00a | 0.01 ± 0.01a |
| *Alishewanella* | 6.40 ± 0.58a | 5.41 ± 0.68a | 3.13 ± 0.14b | 5.85 ± 0.53a |
| *Methylosinus* | 43.72 ± 3.22b | 22.21 ± 10.91bc | 76.78 ± 1.68a | 11.36 ± 1.59c |
| *Methylocystis* | 28.71 ± 1.06b | 37.83 ± 1.04a | 9.94 ± 1.07d | 25.05 ± 1.47b |
| *Methylocapsa* | 3.05 ± 1.57a | 9.74 ± 5.20a | 5.01 ± 1.99a | 8.95 ± 1.76a |
| 20-25 cm | | | | |
| *Rhizobiales* | 88.46 ± 1.15a | 91.34 ± 5.85a | 92.42 ± 2.54a | 45.83 ± 7.70b |
| *Alteromonadales* | 3.61 ± 0.43b | 2.66 ± 0.89bc | 2.52 ± 0.13bc | 6.79 ± 0.93a |
| *Methylococcales* | 4.83 ± 1.87a | 3.53 ± 3.19ab | 0.56 ± 0.52b | 1.11 ± 0.80b |
| *Methylocystaceae* | 75.39 ± 2.34b | 88.47 ± 7.71ab | 91.49 ± 2.96a | 38.63 ± 2.05c |
| *Methylococcaceae* | 0.00 ± 0.00a | 0.00 ± 0.00a | 0.00 ± 0.00a | 0.00 ± 0.00a |
| *Beijerinckiaceae* | 13.07 ± 1.19a | 2.87 ± 1.87b | 0.94 ± 0.46b | 7.20 ± 5.85ab |
| *Alteromonadaceae* | 3.61 ± 0.43b | 2.66 ± 0.89bc | 2.52 ± 0.13bc | 6.79 ± 0.93a |
| *Methylosarcina* | 0.00 ± 0.00a | 0.00 ± 0.00a | 0.00 ± 0.00a | 0.00 ± 0.00a |
| *Methyloparacoccus* | 0.00 ± 0.00a | 0.00 ± 0.00a | 0.00 ± 0.00a | 0.00 ± 0.00a |
| *Candidatus Methylospira* | 0.00 ± 0.00a | 0.00 ± 0.00a | 0.00 ± 0.00a | 0.00 ± 0.00a |
| *Alishewanella* | 3.61 ± 0.43b | 2.66 ± 0.89bc | 2.52 ± 0.13bc | 6.79 ± 0.93a |
| *Methylosinus* | 62.14 ± 0.70b | 78.84 ± 11.92ab | 82.14 ± 4.00a | 12.72 ± 4.11c |
| *Methylocystis* | 12.48 ± 1.66b | 8.42 ± 4.46b | 8.31 ± 1.59b | 24.00 ± 1.54a |
| *Methylocapsa* | 13.07 ± 1.99a | 2.87 ± 1.87b | 0.94 ± 0.46b | 7.20 ± 5.85ab |
| 30-35 cm | | | | |
| *Rhizobiales* | 91.40 ± 2.22a | 96.45 ± 1.27a | 89.30 ± 2.30a | 60.22 ± 10.38b |
| *Alteromonadales* | 2.60 ± 0.14a | 1.62 ± 0.30a | 2.31 ± 0.20a | 4.36 ± 1.05a |
| *Methylococcales* | 2.55 ± 1.84b | 0.27 ± 0.15b | 1.92 ± 1.14b | 18.70 ± 6.12a |
| *Methylocystaceae* | 80.43 ± 2.79b | 93.86 ± 1.22a | 88.07 ± 2.78ab | 52.09 ± 7.88c |
| *Methylococcaceae* | 0.00 ± 0.00a | 0.00 ± 0.00a | 0.11 ± 0.10a | 0.00 ± 0.00a |
| *Beijerinckiaceae* | 10.98 ± 0.59a | 2.58 ± 0.29b | 1.23 ± 0.56b | 8.12 ± 3.90a |
| *Alteromonadaceae* | 2.60 ± 0.14a | 1.62 ± 0.30a | 2.31 ± 0.20a | 4.36 ± 1.05a |
| *Methylosarcina* | 0.00 ± 0.00a | 0.00 ± 0.00a | 0.00 ± 0.00a | 0.00 ± 0.00a |
| *Methyloparacoccus* | 0.00 ± 0.00a | 0.00 ± 0.00a | 0.00 ± 0.00a | 0.00 ± 0.00a |
| *Candidatus Methylospira* | 0.00 ± 0.00b | 0.00 ± 0.00b | 0.11 ± 0.10a | 0.00 ± 0.00b |
| *Alishewanella* | 2.60 ± 0.14a | 1.62 ± 0.30a | 2.31 ± 0.20a | 4.36 ± 1.05a |
| *Methylosinus* | 71.96 ± 2.41b | 89.34 ± 1.39a | 78.10 ± 1.57b | 29.57 ± 8.05c |
| *Methylocystis* | 7.58 ± 0.38b | 3.60 ± 0.21c | 9.03 ± 1.53b | 21.44 ± 6.15a |
| *Methylocapsa* | 10.98 ± 0.59a | 2.58 ± 0.29b | 1.23 ± 0.56b | 8.12 ± 3.90a |
| Taxon | 0-5 cm | 10-15 cm | 20-25 cm | 30-35 cm |
| EHB1 |  |  |  |  |
| *Rhizobiales* | 50.64 ± 0.84c | 77.07 ± 2.29b | 88.46 ± 1.15a | 91.40 ± 2.22a |
| *Alteromonadales* | 5.51 ± 0.28a | 6.40 ± 0.58a | 3.61 ± 0.43b | 2.60 ± 0.14b |
| *Methylococcales* | 29.18 ± 1.33a | 0.47 ± 0.13b | 4.83 ± 1.87b | 2.55 ± 1.84b |
| *Methylocystaceae* | 37.35 ± 1.76b | 74.02 ± 3.79a | 75.39 ± 2.34a | 80.43 ± 2.79a |
| *Methylococcaceae* | 0.00 ± 0.00a | 0.00 ± 0.01a | 0.00 ± 0.00a | 0.00 ± 0.00a |
| *Beijerinckiaceae* | 13.29 ± 1.22a | 3.05 ± 1.57b | 13.07 ± 1.19a | 10.98 ± 0.59a |
| *Alteromonadaceae* | 5.51 ± 0.28a | 6.40 ± 0.58a | 3.61 ± 0.43b | 2.60 ± 0.14b |
| *Methylosarcina* | 0.00 ± 0.00a | 0.00 ± 0.01a | 0.00 ± 0.00a | 0.00 ± 0.00a |
| *Methyloparacoccus* | 0.00 ± 0.00a | 0.00 ± 0.00a | 0.00 ± 0.00a | 0.00 ± 0.00a |
| *Candidatus Methylospira* | 0.00 ± 0.00a | 0.00 ± 0.00a | 0.00 ± 0.00a | 0.00 ± 0.00a |
| *Alishewanella* | 5.51 ± 0.28a | 6.40 ± 0.58a | 3.61 ± 0.43b | 2.60 ± 0.14b |
| *Methylosinus* | 10.71 ± 1.08d | 43.72 ± 3.22c | 62.14 ± 0.70b | 71.96 ± 2.41a |
| *Methylocystis* | 25.49 ± 1.72a | 28.71 ± 1.06a | 12.48 ± 1.66b | 7.58 ± 0.38b |
| *Methylocapsa* | 13.29 ± 1.22a | 3.05 ± 1.57b | 13.07 ± 1.99a | 10.98 ± 0.59a |
| NNF1 |  |  |  |  |
| *Rhizobiales* | 86.92 ± 2.82a | 70.80 ± 13.62a | 91.34 ± 5.85a | 96.45 ± 1.27a |
| *Alteromonadales* | 3.39 ± 0.17a | 5.41 ± 0.68a | 2.66 ± 0.89ab | 1.62 ± 0.30b |
| *Methylococcales* | 5.89 ± 2.04a | 14.94 ± 17.19a | 3.53 ± 3.19a | 0.27 ± 0.15a |
| *Methylocystaceae* | 43.03 ± 6.17b | 61.06 ± 11.90ab | 88.47 ± 7.71a | 93.86 ± 1.22a |
| *Methylococcaceae* | 0.09 ± 0.16a | 0.03 ± 0.05a | 0.00 ± 0.00a | 0.00 ± 0.00a |
| *Beijerinckiaceae* | 43.90 ± 3.39a | 9.74 ± 5.20b | 2.87 ± 1.87b | 2.58 ± 0.29b |
| *Alteromonadaceae* | 3.39 ± 0.17a | 5.41 ± 0.68a | 2.66 ± 0.89ab | 1.62 ± 0.30b |
| *Methylosarcina* | 0.00 ± 0.00a | 0.00 ± 0.00a | 0.00 ± 0.00a | 0.00 ± 0.00a |
| *Methyloparacoccus* | 0.09 ± 0.16a | 0.00 ± 0.00a | 0.00 ± 0.00a | 0.00 ± 0.00a |
| *Candidatus Methylospira* | 0.00 ± 0.00a | 0.03 ± 0.05a | 0.00 ± 0.00a | 0.00 ± 0.00a |
| *Alishewanella* | 3.39 ± 0.17a | 5.41 ± 0.68a | 2.66 ± 0.89ab | 1.62 ± 0.30b |
| *Methylosinus* | 16.72 ± 6.52b | 22.21 ± 10.91b | 78.84 ± 11.92a | 89.34 ± 1.39a |
| *Methylocystis* | 25.92 ± 1.99b | 37.83 ± 1.04a | 8.42 ± 4.46c | 3.60 ± 0.21c |
| *Methylocapsa* | 43.90 ± 3.39a | 9.74 ± 5.20b | 2.87 ± 1.87b | 2.58 ± 0.29b |
| YLC2 |  |  |  |  |
| *Rhizobiales* | 87.18 ± 2.97a | 92.89 ± 0.69a | 92.42 ± 2.54a | 89.30 ± 2.30a |
| *Alteromonadales* | 2.59 ± 0.16b | 3.13 ± 0.14a | 2.52 ± 0.13b | 2.31 ± 0.20b |
| *Methylococcales* | 0.95 ± 0.38a | 1.15 ± 0.62a | 0.56 ± 0.52a | 1.92 ± 1.14a |
| *Methylocystaceae* | 81.63 ± 2.83a | 87.88 ± 1.63a | 91.49 ± 2.96a | 88.07 ± 2.78a |
| *Methylococcaceae* | 0.59 ± 0.21a | 0.00 ± 0.00c | 0.00 ± 0.00c | 0.11 ± 0.10b |
| *Beijerinckiaceae* | 5.55 ± 5.64a | 5.01 ± 1.99a | 0.94 ± 0.46a | 1.23 ± 0.56a |
| *Alteromonadaceae* | 2.59 ± 0.16b | 3.13 ± 0.14a | 2.52 ± 0.13b | 2.31 ± 0.20b |
| *Methylosarcina* | 0.00 ± 0.00a | 0.00 ± 0.00a | 0.00 ± 0.00a | 0.00 ± 0.00a |
| *Methyloparacoccus* | 0.43 ± 0.38a | 0.00 ± 0.00b | 0.00 ± 0.00b | 0.00 ± 0.00b |
| *Candidatus Methylospira* | 0.16 ± 0.17a | 0.00 ± 0.00a | 0.00 ± 0.00a | 0.11 ± 0.10a |
| *Alishewanella* | 2.59 ± 0.16b | 3.13 ± 0.14a | 2.52 ± 0.13b | 2.31 ± 0.20b |
| *Methylosinus* | 72.75 ± 2.80a | 76.78 ± 1.68a | 82.14 ± 4.00a | 78.10 ± 1.57a |
| *Methylocystis* | 7.83 ± 0.13a | 9.94 ± 1.07a | 8.31 ± 1.59a | 9.03 ± 1.53a |
| *Methylocapsa* | 5.55 ± 5.64a | 5.01 ± 1.99a | 0.94 ± 0.46a | 1.23 ± 0.56a |
| YLC6 |  |  |  |  |
| *Rhizobiales* | 30.74 ± 2.07b | 45.99 ± 1.76a | 45.83 ± 7.70a | 60.22 ± 10.38a |
| *Alteromonadales* | 4.40 ± 0.14a | 5.85 ± 0.53a | 6.79 ± 0.93a | 4.36 ± 1.05a |
| *Methylococcales* | 35.90 ± 0.87a | 22.32 ± 2.56b | 1.11 ± 0.80c | 18.70 ± 6.12b |
| *Methylocystaceae* | 21.45 ± 2.61b | 37.04 ± 3.05a | 38.63 ± 2.05a | 52.09 ± 7.88a |
| *Methylococcaceae* | 0.00 ± 0.00a | 0.01 ± 0.01a | 0.00 ± 0.00a | 0.00 ± 0.00a |
| *Beijerinckiaceae* | 9.29 ± 1.41a | 8.95 ± 1.76a | 7.20 ± 5.85a | 8.12 ± 3.90a |
| *Alteromonadaceae* | 4.40 ± 0.14a | 5.85 ± 0.53a | 6.79 ± 0.93a | 4.36 ± 1.05a |
| *Methylosarcina* | 0.00 ± 0.00a | 0.00 ± 0.00a | 0.00 ± 0.00a | 0.00 ± 0.00a |
| *Methyloparacoccus* | 0.00 ± 0.00a | 0.00 ± 0.00a | 0.00 ± 0.00a | 0.00 ± 0.00a |
| *Candidatus Methylospira* | 0.00 ± 0.00 | 0.01 ± 0.01a | 0.00 ± 0.00a | 0.00 ± 0.00a |
| *Alishewanella* | 4.40 ± 0.14a | 5.85 ± 0.53a | 6.79 ± 0.93a | 4.36 ± 1.05a |
| *Methylosinus* | 3.84 ± 1.00b | 11.36 ± 1.59a | 12.72 ± 4.11a | 29.57 ± 8.05a |
| *Methylocystis* | 17.23 ± 1.70b | 25.05 ± 1.47a | 24.00 ± 1.54a | 21.44 ± 6.15ab |
| *Methylocapsa* | 9.29 ± 1.41a | 8.95 ± 1.76a | 7.20 ± 5.85a | 8.12 ± 3.90a |

Values are means ± standard error (n = 3). Lowercase letters in the same row indicate significant difference (α = 0.05) based on Dunn’s multiple comparison tests.

**TABLE S6** The abundance of the *mcrA* and *pmoA* genes and the potential methane production and oxidation at different depths across the four sites at the Dajiuhu Peatland

| Site | Depth  (cm) | *mcrA*  (copies g^-1^ fresh peat) | Type Ia *pmoA*  (copies g^-1^ fresh peat) | Type Ib *pmoA*  (copies g^-1^ fresh peat) | Type II *pmoA*  (copies g^-1^ fresh peat) | PMP  (nmol g^-1^ h^-1^) | PMO  (nmol g^-1^ h^-1^) |
| --- | --- | --- | --- | --- | --- | --- | --- |
| EHB1 | 0 – 5 | 2.60 × 10^7^ (1.76 × 10^6^) | 3.40 × 10^6^ (1.79 × 10^5^) | 6.65 × 10^7^ (5.35 × 10^6^) | 2.20 × 10^7^ (5.81 ×10^5^) | 0.15 (0.03) | 32.62 (2.80) |
|  | 10 – 15 | 6.15 × 10^7^ (8.21 × 10^5^) | 8.15 × 10^5^ (5.16 × 10^4^) | 1.87 × 10^7^ (1.39 × 10^6^) | 2.74 × 10^7^ (2.83 × 10^6^) | 0.35 (0.06) | 19.56 (11.46) |
|  | 20 – 25 | 4.90 × 10^7^ (6.69 × 10^6^) | 3.47 × 10^5^ (5.81 × 10^4^) | 1.42 × 10^7^ (1.04 × 10^6^) | 8.91 × 10^6^ (8.47 ×10^5^) | 0.25 (0.09) | 16.70 (5.19) |
|  | 30 – 35 | 8.56 × 10^6^ (7.87 × 10^5^) | 1.73 × 10^5^ (2.21 × 10^4^) | 8.89 × 10^6^ (2.26 × 10^6^) | 2.64 × 10^6^ (1.75 ×10^5^) | 0.10 (0.01) | 8.13 (2.80) |
| NNF1 | 0 – 5 | 1.91 × 10^5^ (3.14 × 10^4^) | 3.78 × 10^6^ (1.51 × 10^5^) | 5.36 × 10^6^ (1.12 × 10^6^) | 4.81 × 10^7^ (4.56 ×10^6^) | 0.002 (0.001) | 43.09 (9.09) |
|  | 10 – 15 | 3.72 × 10^7^ (7.62 × 10^5^) | 2.91 × 10^6^ (6.16 × 10^5^) | 3.17 × 10^6^ (6.20 × 10^5^) | 5.93 × 10^7^ (7.20 ×10^6^) | 0.032 (0.012) | 35.48 (14.30) |
|  | 20 – 25 | 1.76 × 10^7^ (2.45 × 10^6^) | 4.47 × 10^5^ (1.69 × 10^4^) | 1.96 × 10^6^ (6.42 × 10^4^) | 5.30 × 10^6^ (3.98 ×10^5^) | 0.011 (0.007) | 9.22 (2.73) |
|  | 30 – 35 | 3.75 × 10^6^ (1.79 × 10^5^) | 3.88 × 10^5^ (9.43 × 10^4^) | 9.56 × 10^5^ (7.14 × 10^4^) | 2.00 × 10^6^ (2.85 ×10^4^) | 0.009 (0.004) | 11.79 (1.97) |
| YLC2 | 0 – 5 | 8.26 × 10^6^ (1.06 × 10^5^) | 4.51 × 10^5^ (2.64 × 10^4^) | 7.48 × 10^6^ (1.65 × 10^6^) | 5.61 × 10^6^ (4.22 ×10^5^) | 1.04 (0.44) | 24.34 (17.85) |
|  | 10 – 15 | 3.56 × 10^7^ (3.42 × 10^6^) | 4.39 × 10^5^ (7.34 × 10^4^) | 1.81 × 10^6^ (3.73 × 10^5^) | 9.18 × 10^6^ (1.39 ×10^6^) | 1.24 (0.28) | 18.41 (9.70) |
|  | 20 – 25 | 7.75 × 10^6^ (7.00 × 10^5^) | 4.12 × 10^5^ (3.74 × 10^4^) | 1.50 × 10^6^ (1.15 × 10^5^) | 5.10 × 10^6^ (5.29 ×10^5^) | 0.24 (0.07) | 10.01 (4.57) |
|  | 30 – 35 | 6.05 × 10^6^ (9.47 × 10^5^) | 3.37 × 10^5^ (2.59 × 10^4^) | 6.81 × 10^5^ (1.27 × 10^5^) | 3.38 × 10^6^ (3.53 ×10^5^) | 0.12 (0.03) | 9.09 (0.18) |
| YLC6 | 0 – 5 | 7.50 × 10^7^ (4.96 × 10^6^) | 1.70 × 10^6^ (3.54 × 10^4^) | 1.16 × 10^7^ (9.33 × 10^5^) | 3.69 × 10^7^ (1.46 ×10^6^) | 17.13 (4.97) | 19.44 (3.39) |
|  | 10 – 15 | 8.70 × 10^7^ (5.59 × 10^6^) | 1.59 × 10^6^ (1.28 × 10^5^) | 3.10 × 10^6^ (4.38 × 10^5^) | 3.01 × 10^7^ (4.56 ×10^6^) | 24.53 (9.11) | 16.46 (3.55) |
|  | 20 – 25 | 6.43 × 10^7^ (3.60 × 10^6^) | 1.27 × 10^6^ (2.57 × 10^5^) | 2.38 × 10^6^ (2.32 × 10^4^) | 1.54 × 10^7^ (1.24 ×10^6^) | 5.24 (2.07) | 7.43 (3.17) |
|  | 30 – 35 | 2.41 × 10^7^ (2.37 ×10^6^) | 4.19 × 10^5^ (4.04 × 10^4^) | 8.69 × 10^5^ (7.55 × 10^4^) | 5.12 × 10^6^ (8.63 ×10^5^) | 2.22 (0.70) | 6.10 (0.25) |

PMP, potential methane production; PMO, potential methane oxidation. NNF1, the first sampling site of Niangniangfen; YLC2, the second sampling site of Yangluchang; YLC6, the sixth sampling site of Yangluchang. Values in parentheses represent standard errors (n = 3 for qPCR, n = 6 for PMP and PMO).

**TABLE S7** Modified normalized stochasticity ratio (MST) of methanogenic and methanotrophic communities across the four sites in peat sediments

| Category | Methanogenic community | | | Methanotrophic community | | |
| --- | --- | --- | --- | --- | --- | --- |
|  | MST (%) | Stochastic processes | Deterministic processes | MST (%) | Stochastic processes | Deterministic processes |
| EHB1 | 33.17 | 33.33% | 66.67% | 46.13 | 54.55% | 45.45% |
| NNF1 | 48.51 | 50.00% | 50.00% | 39.61 | 30.30% | 69.70% |
| YLC2 | 44.08 | 34.85% | 65.15% | 37.91 | 13.64% | 86.36% |
| YLC6 | 48.06 | 42.42% | 57.58% | 61.96 | 72.73% | 27.27% |
| Average | 43.45 | 40.15% | 59.85% | 46.40 | 42.80% | 57.20% |

**TABLE S8** Levin’s niche breadth of methanogenic and methanotrophic communities across the four sites in peat sediments

| Category | Levin’s niche breadth | |
| --- | --- | --- |
|  | Methanogenic community | Methanotrophic community |
| EHB1 | 2.66 ± 1.77b | 3.39 ± 2.47a |
| NNF1 | 4.03 ± 2.26a | 2.96 ± 2.02b |
| YLC2 | 3.32 ± 2.32a | 2.44 ± 1.88b |
| YLC6 | 3.56 ± 2.41a | 3.64 ± 2.47a |
| Whole | 5.84 ± 3.74b | 6.63 ± 5.07a |

Lowercase letters in the same row indicate significant difference (α = 0.05) based on Mann-Whitney U tests.


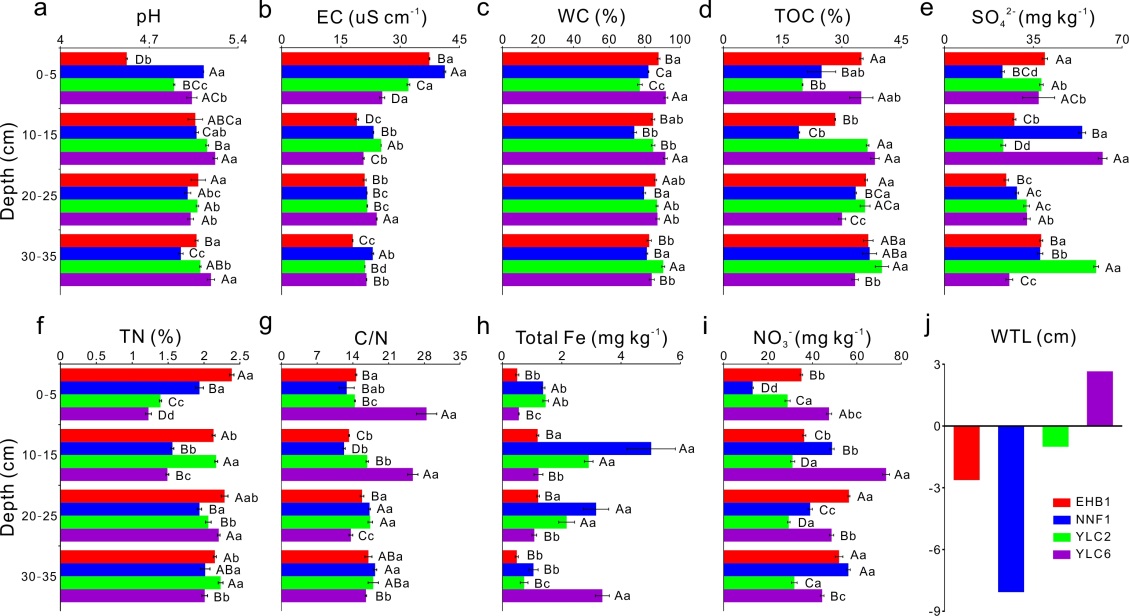


**FIG S1** Physicochemical properties of peat sediments across depths and sites. (a) pH; (b) EC, electrical conductivity; (c) WC, water content; (d) TOC, total organic carbon; (e) Sulfate; (f) TN, total nitrogen; (g) C/N, the ratio of total organic carbon to total nitrogen; (h) Total Fe; (i) Nitrate; (j) WTL, water table level. Values are mean ± standard error (n = 3). Capital letters mean a statistical significance (*P* < 0.05) among four sites within the same depth, and small letters mean a statistical significance (*P* < 0.05) among different depths with one site. EHB1, the first site of Erhaoba; NNF1, the first site of Niangniangfen; YLC2, the second site of Yangluchang; YLC6, the sixth site of Yangluchang. The WTL is expressed with negative number when it is below the peat surface and vice versa.


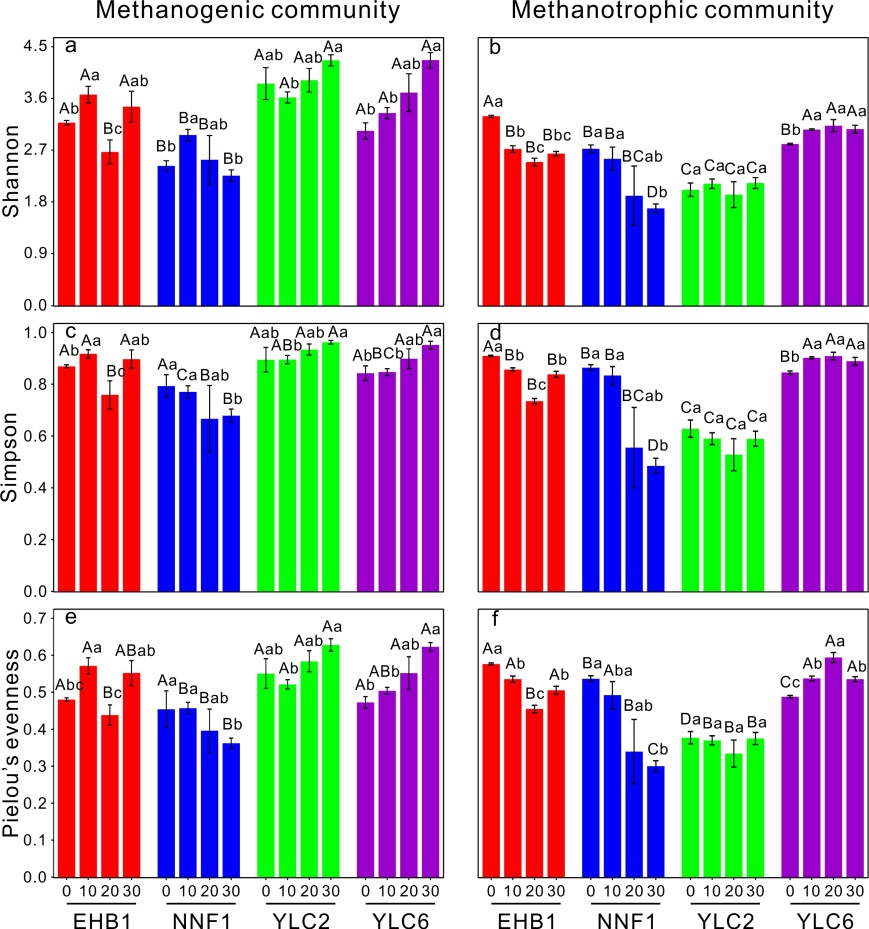


**FIG S2** Alpha diversity of methanogens (a, c, e) and methanotrophs (b, d, f) from peat sediments across depths and sites. The number 0, 10, 20 and 30 on the x-axis represent the depth of 0 - 5, 10 - 15, 20 - 25 and 30 - 35 cm, respectively, among four sites. The letters and abbreviations are listed in the Fig. S1.


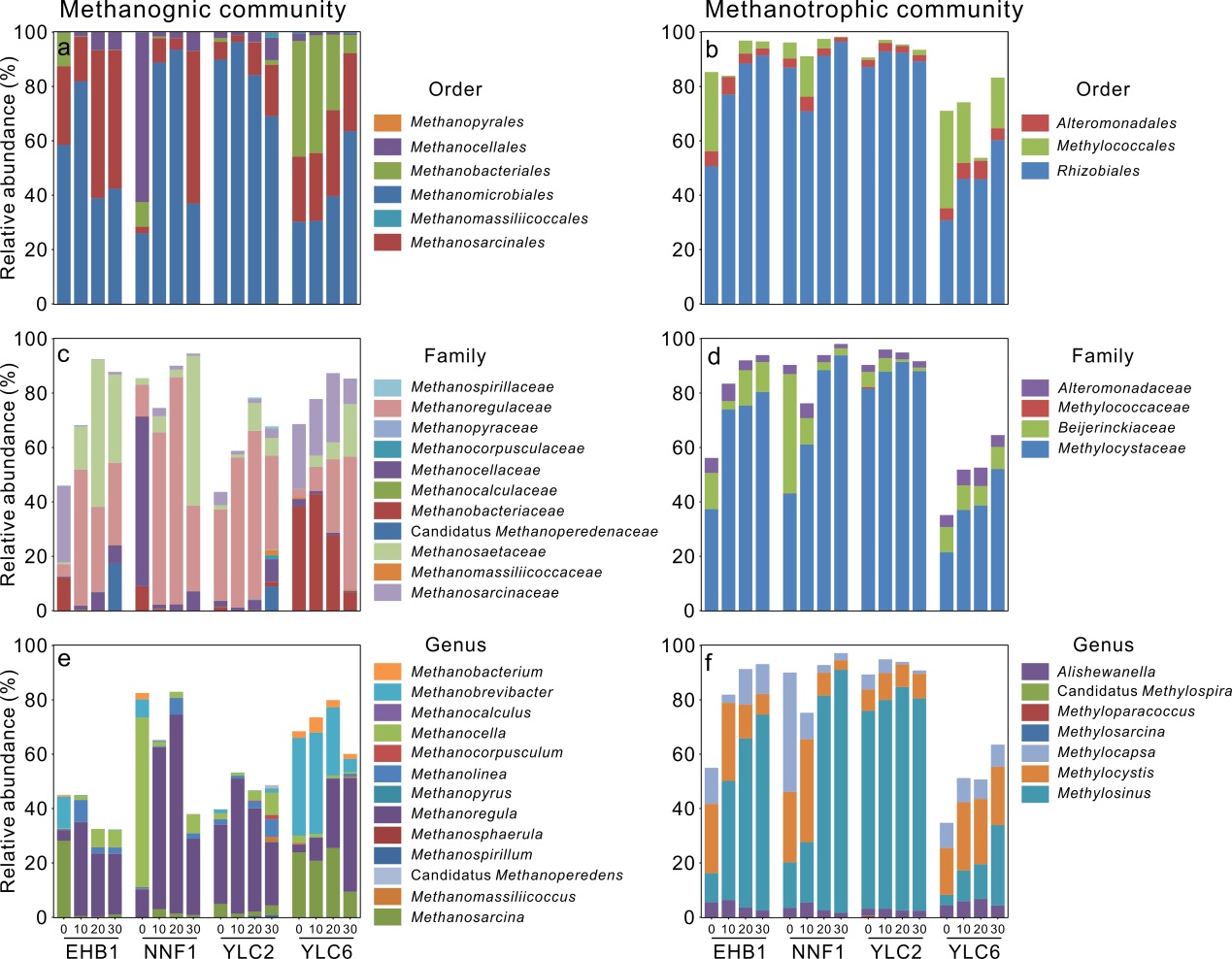


**FIG S3** Composition of methanogens (a, c, e) and methanotrophs (b, d, f) at the order, family and genus levels from peat sediments across depths and sites. The number 0, 10, 20 and 30 on the x-axis are the same as indicated by Fig. S2.


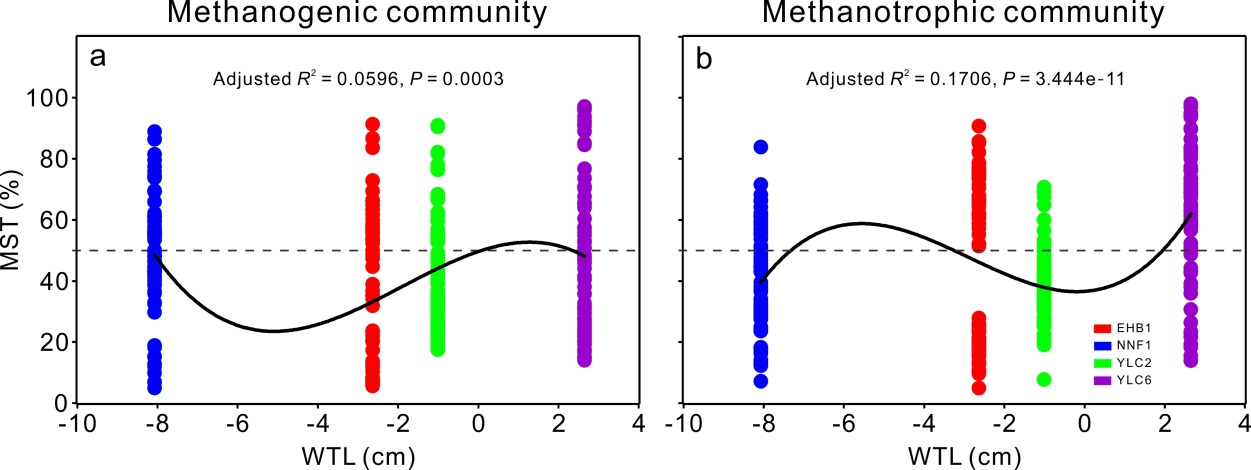


**FIG S4** Variations in modified normalized stochasticity ratio (MST) for methanogenic (a) and methanotrophic (b) communities with water table. WTL, water table level.


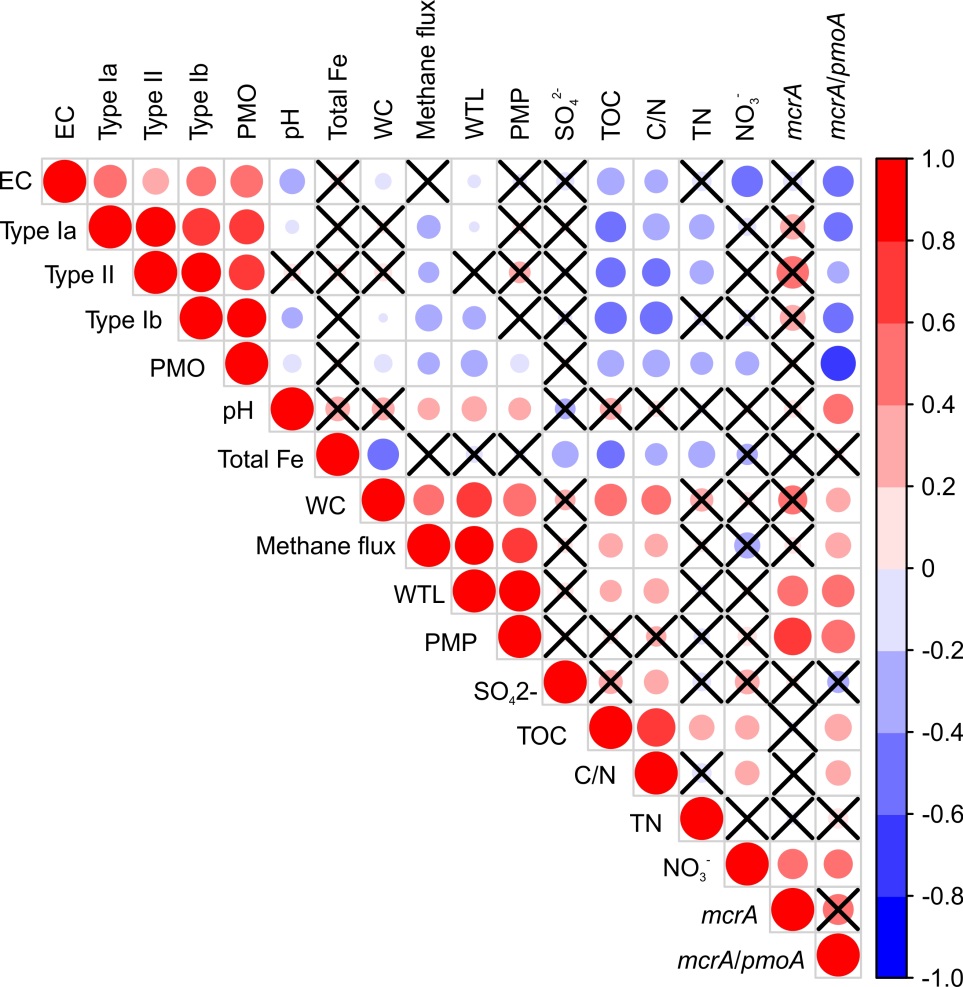


**FIG S5** Correlation between environmental factors and the characteristic of methane microbial communities. The heatmap depicts Pearson correlation where statistically significantly (*P* < 0.05) positive correlations (red), statistically significantly (*P* < 0.05) negative correlations (blue), and a lack of a significant correlation is marked by a cross. PMO, potential methane oxidation; PMP, potential methane production. Other abbreviations are referred to the Fig. S1.
